# A signature of epithelial-mesenchymal plasticity and stromal activation in primary tumor modulates late recurrence in breast cancer independent of disease subtype

**DOI:** 10.1186/s13058-014-0407-9

**Published:** 2014-07-25

**Authors:** Qing Cheng, Jeffrey T Chang, William R Gwin, Jun Zhu, Stefan Ambs, Joseph Geradts, H Kim Lyerly

**Affiliations:** 1 Department of Surgery, Duke University Medical Center, 203 Research Drive, Durham, 27710 NC USA; 2 Department of Integrative Biology and Pharmacology, University of Texas Health Science Center, 6431 Fannin Street, Houston, 77030 TX USA; 3 Department of Medicine, Duke University Medical Center, 101B MSRB, Durham, 27710 NC USA; 4 Systems Biology Center, National Heart, Lung and Blood Institute, National Institutes of Health, 9000 Rockville Pike, Bethesda, 20892 MD USA; 5 Laboratory of Human Carcinogenesis, National Cancer Institute, National Institutes of Health, 9000 Rockville Pike, Bethesda, 20892 MD USA; 6 Department of Pathology, Duke University Medical Center, 3108 Meyer Ward, Durham, 27710 NC USA

## Abstract

**Introduction:**

Despite improvements in adjuvant therapy, late systemic recurrences remain a lethal consequence of both early- and late-stage breast cancer. A delayed recurrence is thought to arise from a state of tumor dormancy, but the mechanisms that govern tumor dormancy remain poorly understood.

**Methods:**

To address the features of breast tumors associated with late recurrence, but not confounded by variations in systemic treatment, we compiled breast tumor gene expression data from 4,767 patients and established a discovery cohort consisting of 743 lymph node-negative patients who did not receive systemic neoadjuvant or adjuvant therapy. We interrogated the gene expression profiles of the 743 tumors and identified gene expression patterns that were associated with early and late disease recurrence among these patients. We applied this classification to a subset of 46 patients for whom expression data from microdissected tumor epithelium and stroma was available, and identified a distinct gene signature in the stroma and also a corresponding tumor epithelium signature that predicted disease recurrence in the discovery cohort. This tumor epithelium signature was then validated as a predictor for late disease recurrence in the entire cohort of 4,767 patients.

**Results:**

We identified a novel 51-gene signature from microdissected tumor epithelium associated with late disease recurrence in breast cancer independent of the molecular disease subtype. This signature correlated with gene expression alterations in the adjacent tumor stroma and describes a process of epithelial to mesenchymal transition (EMT) and tumor-stroma interactions.

**Conclusions:**

Our findings suggest that an EMT-related gene signature in the tumor epithelium is related to both stromal activation and escape from disease dormancy in breast cancer. The presence of a late recurrence gene signature in the primary tumor also suggests that intrinsic features of this tumor regulate the transition of disseminated tumor cells into a dormant phenotype with the ability to outgrowth as recurrent disease.

**Electronic supplementary material:**

The online version of this article (doi:10.1186/s13058-014-0407-9) contains supplementary material, which is available to authorized users.

## Introduction

Delayed recurrence, common in breast cancer, is defined as the clinical appearance of cancer systemically or locally years (five to twenty-five years) after eradication of the primary tumor and adjuvant therapy in a patient who has been clinically disease-free [1]. While patients who have distant or regional metastases at the time of diagnosis have predictably poor clinical outcomes including death from breast cancer, other patients diagnosed at an early stage, with small tumors and no evidence of regional lymph node metastases, can have a late systemic recurrence, occurring in as many as one-third of patients if followed for greater than 10 to 15 years [2]-[4]. While systematic cytotoxic or endocrine therapy after curative local treatment is designed to eradicate occult micrometastases, these therapies typically reduce metastatic recurrences by only a third at 10 years [5]-[8]. The consequences of systemic recurrence are profound, as patients with recurrent breast cancer usually die of their disease despite second- or third-line systemic therapies [9],[10]. The long interval between treatment and recurrence is inconsistent with a model of continuous growth of cancer cells [11]-[15], but instead suggests a state of tumor dormancy [16]. However, mechanisms that allow for, or lead to, tumor dormancy remain very poorly understood and require further study [4],[17]-[20].

With the intention to predict the risk of disease recurrence, several commercially available multigene prognostic assays have been developed, such as Oncotype DX™ [21], PAM50 Breast Cancer Intrinsic Classifier™ [22] and MammaPrint™ [23]. However, none of these predictors was designed to classify patients based on their likelihood of developing a late recurrent disease, as the vast majority of recurrent cases for the development and testing of these predictors had recurrence within five years after initial treatment. In this study, we established a discovery cohort of primary tumors from lymph node-negative patients who did not receive systemic neoadjuvant or adjuvant therapy, and conducted a series of recurrence-free survival analyses to detect differences between late recurrences (recurrence appeared at or after five years), which may be due to tumor dormancy features, and early recurrences (recurrence appeared within five years), which may be due to aggressive tumor invasion and metastasis features. We found a distinct set of genes that modulate either early or late recurrence in breast cancer. Moreover, late recurrences were associated with a gene expression signature in the primary tumor consistent with epithelial to mesenchymal plasticity and the occurrence of tumor-stroma interactions. Lastly, we identified a 51-gene classifier with these characteristics that was significantly associated with late distant recurrence in an independent cohort of 4,767 breast tumor samples. Our results highlight the importance of analyzing the microenvironment of primary tumors for biomarker discovery, and to obtain new insights into the processes that govern breast cancer dormancy.

## Methods

### Develop a 4,769 primary breast cancer expression data set

Our previous study developed an approach to compile a large collection of publicly available gene expression data [24]. To update this data set, we added 759 additional samples of clinical outcome data that was available, and rebuilt this data set. A total of 4,767 breast cancer gene expression profiles were collected from 25 independent data sets (GSE11121, GSE12093, GSE12276, GSE1456, GSE16391, GSE16446, GSE17705, GSE17907, GSE19615, GSE2034, GSE20685, GSE21653, GSE22035, GSE22093, GSE23177, GSE23720, GSE25066, GSE26639, GSE3494, GSE4922, GSE5327, GSE5460, GSE6532, GSE7390, GSE9195) that were on the National Center for Biotechnical Information (NCBI) Gene Expression Omnibus (GEO; Additional file 1).

Primary breast tumor samples were obtained before treatment and gene expression profiles were measured using Affymetrix U133A or U133 Plus 2.0 expression array (Affymetrix, Santa Clara, CA, USA). As we described previously [24], all data were filtered to include those probes on the HG-U133A platform. Assuming that the signal from the 69 Affymetrix control probes should be invariant, we found the structure in those probes by taking the first 40 principal components, and then removed the contribution of those patterns in the expression of genes using Bayesian Factor Regression Modeling (BFRM) [25]. A Principal Component Analysis (PCA) and Heatmap were used to confirm data set normalization.

By fitting two normal distributions of mRNA expression into immunohistochemistry (IHC) positive and negative groups, we identified bimodal cutoff that represents the maximum likelihood of IHC status, using samples where the expression status of human epidermal growth factor receptor 2 (HER2) (n = 1,579), estrogen receptor (ER) (n = 3,918) and progesterone receptor (PR) (n = 2,060) were available [24], and then applied this predictive cutoff to the samples for which the IHC status of HER2, ER and PR was not available. For the samples for which IHC status was available, the final calls for HER2, ER and PR status were defined according to IHC measurement. For the samples for which IHC status was not available, the final calls for HER2, ER and PR status were defined using mRNA expression bimodal cutoffs [24]. Luminal A subtype was defined as ER + and/or PR+, HER2-; luminal B subtype was defined as ER + and/or PR+, HER2+; triple-negative breast cancer (TNBC) was defined as ER-, PR-, HER2-; and HER2 type was defined as ER-, PR-, HER2 + .

### Develop a collection of multi-tissue expression datasets

We developed a data set of 1,042 gene expression profiles from breast tumors, tumor-adjacent stroma, and ductal carcinoma *in situ* (DCIS) from nine independent data sets (GSE2034, GSE4922, GSE6532, GSE7390, GSE5847, GSE3893, GSE16873, GSE21422, GSE19615). In this data set, a total of 763 primary tumor samples obtained from patients who were not treated with systemic neoadjuvant or adjuvant treatment were collected from GSE2034 (286 samples), GSE4922 (142 samples), GSE6532 (137 samples), and GSE7390 (198 samples). Gene expression profiles of both tumor epithelium and matched stromal tissue were obtained from GSE5847 (95 samples). Three data sets (GSE3893 (10 samples), GSE16873 (40 samples), and GSE21422 (19 samples) that contained gene expression profiles of DCIS were also included in this multi-tissue expression data set, though gene expression of DCIS was excluded in this study. This multi-tissue expression data set also contained GSE19615 (115 samples), and the IHC measured status of HER2, ER and PR from GSE19615 was used to select the bimodal cutoff that represents the maximum likelihood of IHC status. In this data set, we revealed the structure in 69 Affymetrix control probes by taking the first 15 principal components, and then removed the contribution of those patterns in the expression of genes using BFRM [25].

### Statistical analyses

In addition to the raw expression data, we also obtained available clinical outcome data from the GEO database, including recurrence-free survival (the events of both local and distant recurrence) and distant metastasis-free survival (the events of first distant metastasis and distant recurrence). A genome-scale Cox regression survival analyses was performed using a total of 11,761 known genes (18,750 probe sets), as described in our previous study [24]. Gene expression signal was used as continuous variable, and co-efficiency was applied to determine if gene expression *per se* was a direct (overexpression was associated with poor outcome) or inverse (overexpression was correlated with good outcome) correlation. For the recurrence-free survival or distant metastasis-free survival analyses, patients’ data was censored by the time of the last follow-up. For the late recurrence-free survival or late distant metastasis-free survival analyses, patients’ data was censored by the time of the last follow-up, or the time of recurrence or metastasis event appearing within five years. For the early recurrence-free survival or early distant metastasis-free survival analyses, patients’ data was censored by the time of the last follow-up, or the time of recurrence appearing at or after five years.

To assess if the correlation between gene expression and prognosis was a truly independent prognostic factor, we conducted an additional genome-scale Cox Proportional-Hazards Regression (COXPH) survival analyses to quantify the weight of the hazard ratios associated with high expression and their significance when considered alongside other clinical variables such as size, grade, nodal status, age, HER2, ER and PR, in the whole cohort or in the relevant subtype of tumors.

Statistical analyses were performed using STATISTICA 11 (Statsoft Inc. Tulsa, OK, USA); R Project for Statistical Computing (Augasse, Austria); Matlab (Natick, MA, USA); GraphPad (La Jolla, CA, USA). Two-way hierarchical clustering was performed using Cluster 3.0 (Tokyo, Japan), and the visualization of microarray data was carried out using Java Treeview (Boston, MA, USA). Pathway analysis was conducted using MetaCore (Thomson Reuters, New York, NY, USA) and Gene Set Enrichment Analysis (GSEA) [26],[27].

## Results and discussion

### Distinct characteristics in primary breast tumors with early or late recurrence

**Figure 1:**
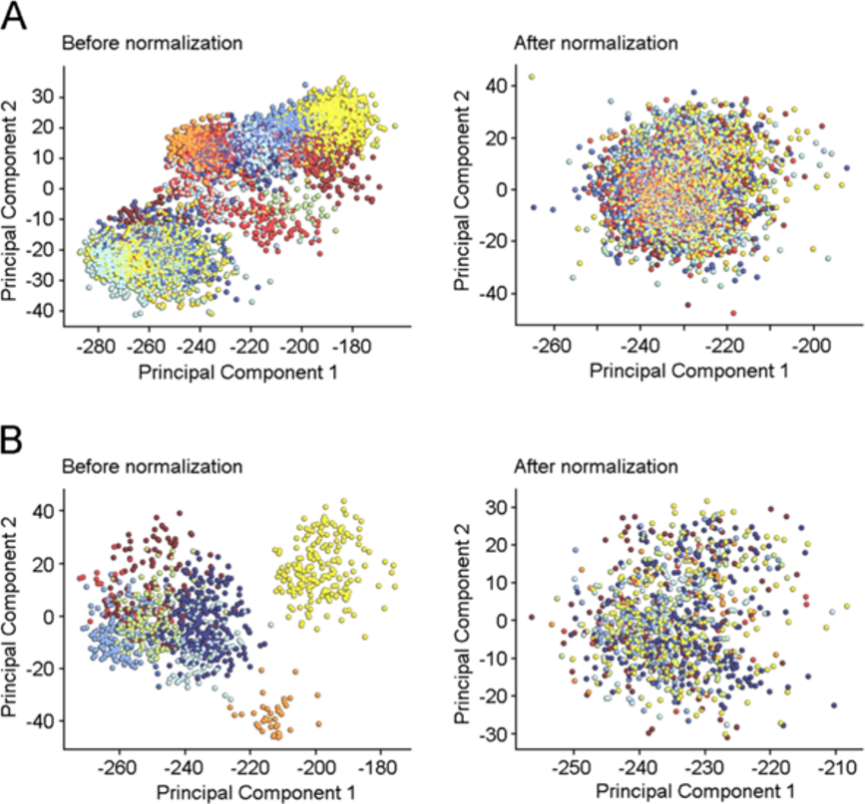
**Development of combined data sets. (A)** PCA plots of 4,767 expression dataset. **(B)** PCA plots of multi-tissue expression dataset. These plots show the gene expression profiles of the samples plotted on the first two principal components. Each point represents a sample, and samples from the same data set have the same color. If there are batch effects, the samples from the same data set (the same color) will cluster together. If there are no batch effects, the colors should be mixed.

**Figure 2:**
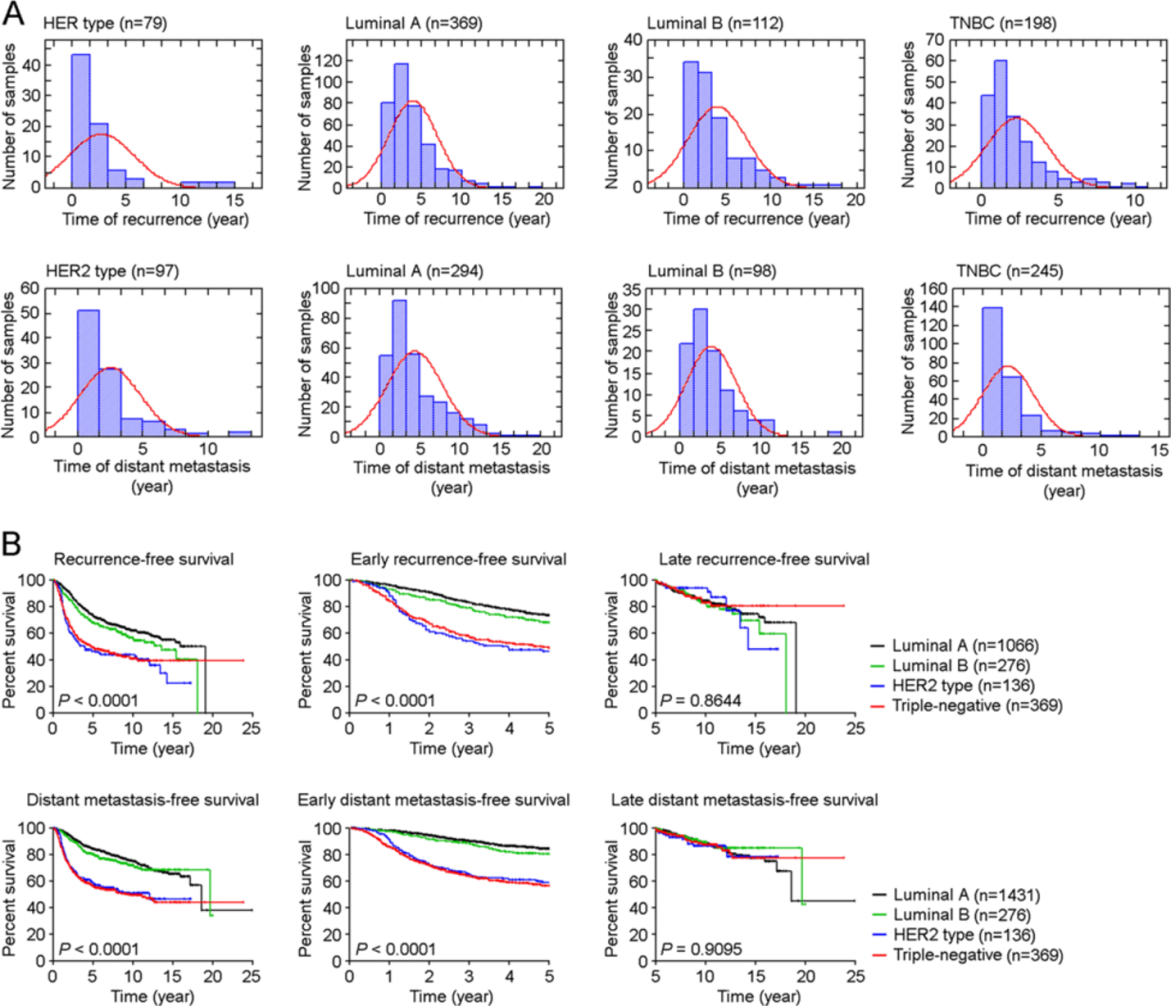
**Correlation between breast cancer molecular subtypes and early or late recurrence. (A)** Histograms of recurrence or distant metastasis events distribution in different breast cancer subtypes among 4,767 samples. **(B)** Subtype difference in overall, early or late recurrence-free survival (n = 1,847) or distant metastasis-free survival (n = 2,612). For the late recurrence-free survival analyses, patients’ data was censored by the time of last follow-up or death, or the time of recurrence appearing within five years. For the early recurrence-free survival analyses, patients’ data was censored by the time of last follow-up or death, or the time of recurrence appearing at/after five years.

Because clinical dormancy has been defined as the time (five to twenty-five years) between removing the primary tumor and relapse [1], we defined late recurrence as recurrence (either local or distant recurrence) that occurred five or more years after initial treatment, and used this clinical scenario as a model to characterize genetic factors that lead to tumor dormancy. We compiled a collection of breast tumor gene expression data (n = 4,767) derived from 25 data sets that were posted on the NCBI GEO database, using the methods that we previously reported [24] (Figure 1; Additional files 1 and 2). We found the greatest risk for early recurrence or distant metastasis in patients with HER2-amplified and TNBC tumors, which led to the poorest recurrence-free survival and distant metastasis-free survival (Figure 2A). However, there was no significant difference for late recurrences or distant metastasis, and late recurrence and distant metastasis occurred in each of molecular disease subtypes (Figure 2B).

To characterize the features of tumors associated with late recurrence not influenced by variations in treatment, we focused on a subset of lymph node-negative breast tumor samples obtained from 743 patients who did not receive systemic neoadjuvant or adjuvant treatment, and found those well-known clinical predictors, such as grade, tumor size and basal-like molecular subtype were significantly associated with early recurrence, but not late recurrence (Table 1). The absence of differences between the various molecular subtypes in this analysis suggested that the phenotype of late recurrence is likely a common phenomenon applicable to all subtypes.

**Table 1:** **Correlation between clinical parameters and early or late recurrence in 743 dataset**

Clinical parameter		N	Early recurrence			Late recurrence		
			***P***value	HR (95% CI)	N (event)	***P***value	HR (95% CI)	N (event)
**HER2**	pos. vs. neg.	743	0.1378	1.36 (0.91 - 2.04)	207	0.7788	1.10 (0.57 - 2.13)	80
**ER**	pos. vs. neg.	743	0.0048	0.63 (0.46 - 0.87)	207	0.5023	1.19 (0.72 - 1.97)	80
**PR**	pos. vs. neg.	743	0.0004	0.61 (0.46 - 0.80)	207	0.1027	1.45 (0.93 - 2.28)	80
**TNBC**	TN vs. others	743	0.0031	1.77 (1.21 - 2.59)	207	0.0974	0.61 (0.34 - 1.10)	80
**Tumor size**	≥2 cm vs. <2 cm	456	0.0008	1.91 (1.31 - 2.78)	114	0.3184	1.29 (0.78 - 2.12)	67
**Grade**	Grade 3 vs. others	438	0.0038	1.87 (1.22 - 2.84)	110	0.0692	0.60 (0.35 - 1.04)	61
**Age**	≥50 vs. <50	457	0.9933	1.00 (0.69 - 1.44)	114	0.1144	0.67 (0.41 - 1.10)	66

CI: confidence interval; ER: estrogen receptor; HER2: human epidermal growth factor receptor 2; HR, hazard ratio; PR: progesterone receptor; TNBC: triple-negative breast cancer.

**Figure 3:**
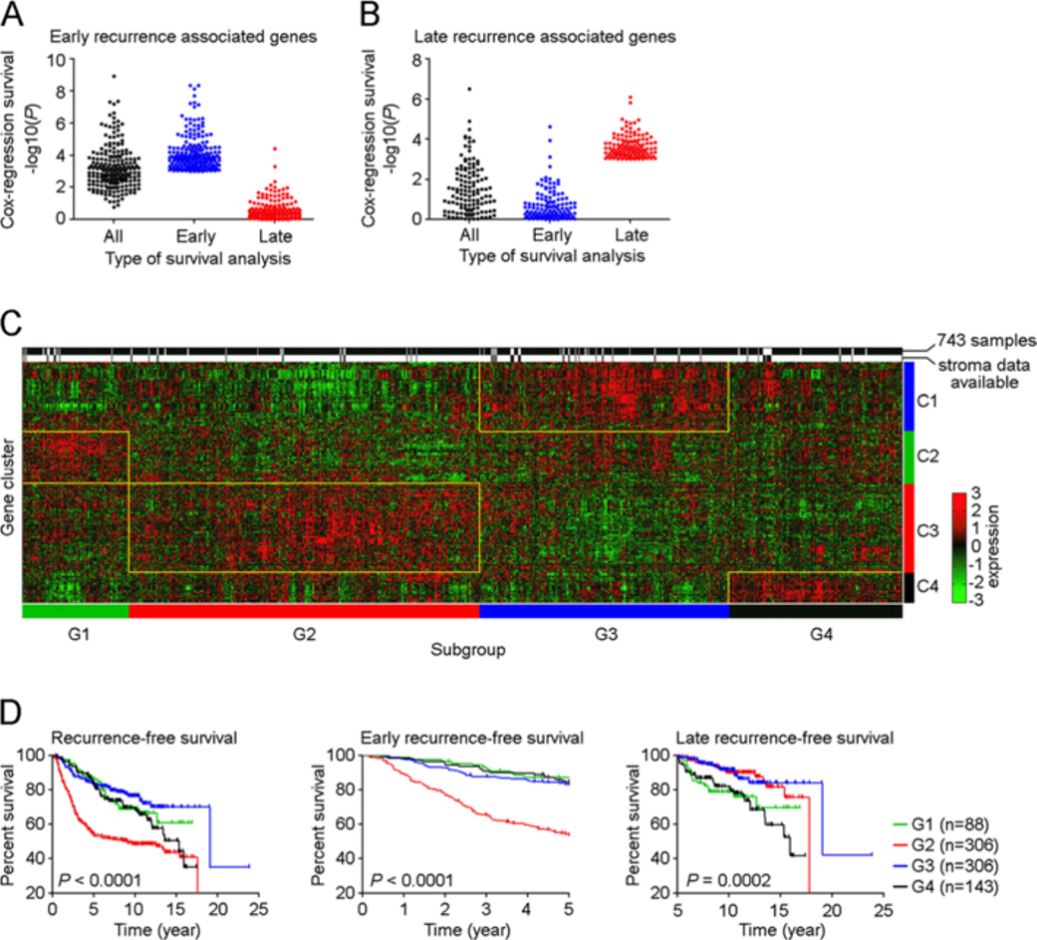
**Analysis of patterns of early or late recurrence-associated genes to define four distinct subgroups of breast cancer. (A)** A total of 208 late recurrence-associated genes (*P* <0.0001) were selected for overall, early and late Cox regression survival analyses, and *P* values from three types of survival analyses are shown. **(B)** A total of 124 late recurrence-associated genes (*P* <0.0001) were selected for overall, early and late Cox regression survival analyses, and *P* value from these three types of survival analyses are shown. **(C)** Two-way hierarchical clustering (Centroid Linkage) of 216 probe sets that were significantly correlated with either early or late recurrence among 789 breast cancer samples, which included 743 lymph node-negative breast tumor samples obtained from patients who did not receive systemic neoadjuvant or adjuvant treatment, and 46 breast tumor epithelium samples for which gene expression of matched stromal tissue were available. Yellow boxes indicate upregulated gene cluster (C1 to C4) in related subgroups (G1 to G4). **(D)** Total, early or late recurrence-free survival was stratified according to breast cancer subgroups (G1 to G4). *Tick marks* in Kaplan-Meier estimates distant-metastasis-free survival indicate patients whose data were censored. *P* values were calculated using log-rank (Mantel-Cox) test.

To assess the biological differences in primary tumors with either early or late recurrence, we developed a multi-tissue gene expression data set (Figure 1; Additional file 2). Using the gene expression data of those 743 samples, we revealed a distinct set of 216 probe sets (189 genes) whose expression was associated with either early or late recurrence (*P* <0.001, n = 743, Cox-regression survival analysis; Figure 3A), and this association was not affected by clinical variables such as size, grade, nodal status, age, HER2, ER and PR status (*P* <0.01, n = 438, COXPH) (Additional file 3).

### High degree of epithelial-mesenchymal plasticity in primary breast tumor was correlated with late recurrence

**Figure 4:**
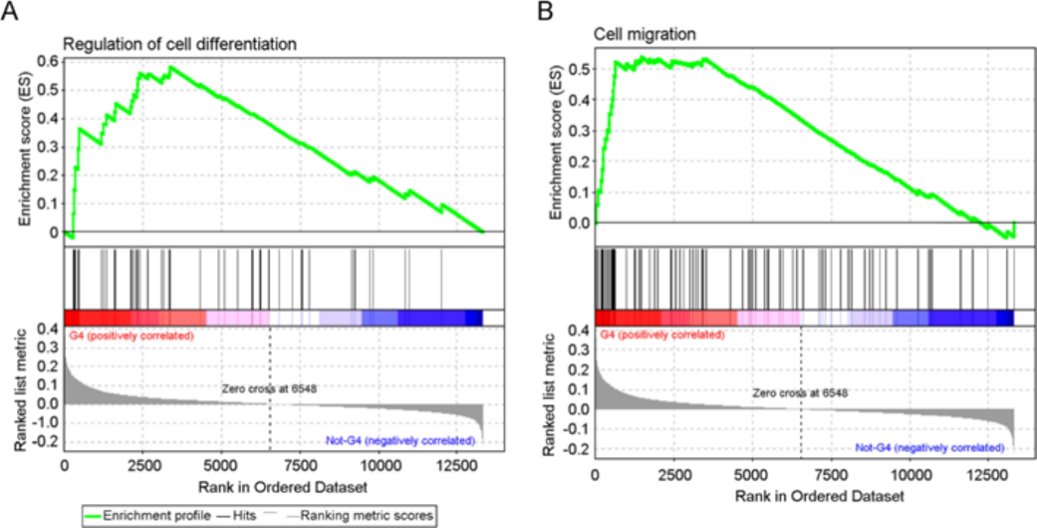
**Gene Set Enrichment Analysis (GSEA) enrichment plot in subgroup G4 versus other subgroups. (A)** GSEA plot for pathways involved in regulation of cell differentiation. **(B)** GSEA plot for pathways involved in cell migration.

Using the 216 probe set classifier, a two-way hierarchical clustering (Centroid Linkage) among those 743 lymph node-negative breast tumor samples was conducted, which revealed multiple subgroups with distinct prognostic characteristics (Figure 3). We found that subgroup G4 was associated with late recurrence (Figure 3), but none of the breast cancer subtypes was significantly enriched nor underrepresented in this subgroup (Table 2). Interestingly, we found *TWIST1*, a key regulator of EMT [28]-[30], was significantly associated with early recurrence (*P* = 1.92 × 10^−4^, COXPH; Additional file 3). However, in subgroup G4 patients, *TWIST1* was co-upregulated with a group of late recurrence-associated genes (cluster C4), and the collective effect of this gene cluster was significantly associated with late recurrence (*P* = 1.48 × 10^−11^, Fisher’s exact test; Table 3). Pathway analysis of gene cluster C4 revealed both transforming growth factor beta (TGFβ)-dependent induction of EMT pathway (objects: *TWIST1*[28]-[30], *JAG1*[31]) and human growth factor (HGF)-dependent inhibition of TGFβ-induced EMT signaling (objects: *HGF*[32]-[35]) were activated in a sample of this subgroup. To access protein functional process of these EMT-related signaling this subgroup G4, we carried out GSEA and found both ‘Regulation of cell differentiation’ and ‘Cell migration’ gene sets were enriched in a sample from subgroup G4, compared with samples in other subgroups (Figure 4). Collectively, results suggested that the tumors from subgroup G4 had a high degree of epithelial-mesenchymal plasticity. Since the EMT state has been associated with quiescence or reduced proliferation [36],[37], the tumor cells with a high degree of epithelial-mesenchymal plasticity could escape from the primary tumor in a (semi)-mesenchymal and stem-like state, and could establish a metastasis at the distant site by reverting to their epithelial phenotype [38],[39].

**Table 2:** **Distribution of breast cancer subtypes in different subgroups**

Subgroup	Subtype	N	Percentage (%)	Fisher’s exact test (***P***value)	Odds ratio (OR)	95% confidence interval (95% CI)
**G1**	**Luminal A**	85	96.5	4.90E-11	14.3244	4.4797 - 45.8043
	**Luminal B**	1	1.1	0.0143	0.1324	0.0181 - 0.9693
	**HER2 type**	1	1.1	0.0329	0.1574	0.0214 - 1.1559
	**TNBC**	1	1.1	1.12E-06	0.0497	0.0069 - 0.3602
**G2**	**Luminal A**	192	62.5	3.88E-04	0.5614	0.4105 - 0.7678
	**Luminal B**	30	9.8	0.0315	1.8715	1.0778 - 3.2496
	**HER2 type**	22	7.2	0.3592	1.3339	0.7378 - 2.4115
	**TNBC**	63	20.5	0.0234	1.5571	1.0631 - 2.2806
**G3**	**Luminal A**	144	63.7	0.0195	0.6664	0.4788 - 0.9275
	**Luminal B**	16	7.1	0.6541	0.844	0.461 - 1.5453
	**HER2 type**	17	7.5	0.6311	1.1713	0.6317 - 2.1719
	**TNBC**	49	21.7	0.1185	1.3737	0.9225 - 2.0456
**G4**	**Luminal A**	113	79	0.0085	1.7894	1.1567 - 2.7682
	**Luminal B**	8	5.6	0.4771	0.7237	0.3342 - 1.5673
	**HER2 type**	7	4.9	0.5674	0.7476	0.3278 - 1.705
	**TNBC**	15	10.5	0.0255	0.5268	0.2972 - 0.9338

CI: confidence interval; HER2: human epidermal growth factor receptor 2; TNBC: triple-negative breast cancer.

**Table 3:** **Distribution of early or late recurrence-associated genes in different gene clusters**

Cluster	Genes associated with	Correlation	Fisher’s exact test (***P***value)	Odds ratio (OR)	95% confidence interval (95% CI)
**C1**	Early recurrence	direct	1.19 × 10^−14^	*no probe set in C1*	
		inverse	2.41 × 10^−24^	89.15	20.82 - 381.84
	Late recurrence	direct	0.0002	0.06	0.02 - 0.48
		inverse	0.1872	0.21	0.03 - 1.65
**C2**	Early recurrence	direct	0.3475	0.41	0.09 - 1.92
		inverse	8.81 × 10^−7^	5.96	2.77 - 12.83
	Late recurrence	direct	0.6402	1.28	0.53 - 3.08
		inverse	0.0744	*no probe set in C2*	
**C3**	Early recurrence	direct	3.05 × 10^−26^	39.14	17.42 - 87.95
		inverse	0.0468	0.02	0.01 - 0.05
	Late recurrence	direct	0.0057	0.27	0.10 - 0.73
		inverse	9.41 × 10^−05^	21.52	2.72 - 170.15
**C4**	Early recurrence	direct	1	0.98	0.42 - 2.30
		inverse	1.50 × 10^−08^	*no probe set in C4*	
	Late recurrence	direct	1.48 × 10^−11^	25.00	9.50 - 65.80
		inverse	0.3697	*no probe set in C4*	

CI: confidence interval.

The significantly higher risk of overall and early recurrence in subgroup G2 was correlated with a paucity of luminal A subtype tumors in this subgroup (Table 2), and an upregulated gene cluster (C3) enriched with genes that were directly correlated with early recurrence or inversely associated with late recurrence (*P* <0.0001, Fisher’s exact test; Table 3). Consistent with our previous finding [24], activated stress response signaling (network object *HSP90AA1*) in gene cluster C3 was correlated with higher risk of early recurrence (Additional files 3 and 4).

Although patients in subgroup G1 experienced increased risk of late recurrence (Figure 3), this subgroup was not considered as a desired model for this study, because subgroup G1 showed an unbalanced distribution of breast cancer molecular subtypes (Table 2), and the upregulated gene cluster C2 was not directly correlated with late recurrence (Table 3).

### An activated microenvironment in primary breast tumor was associated with late recurrence

**Figure 5:**
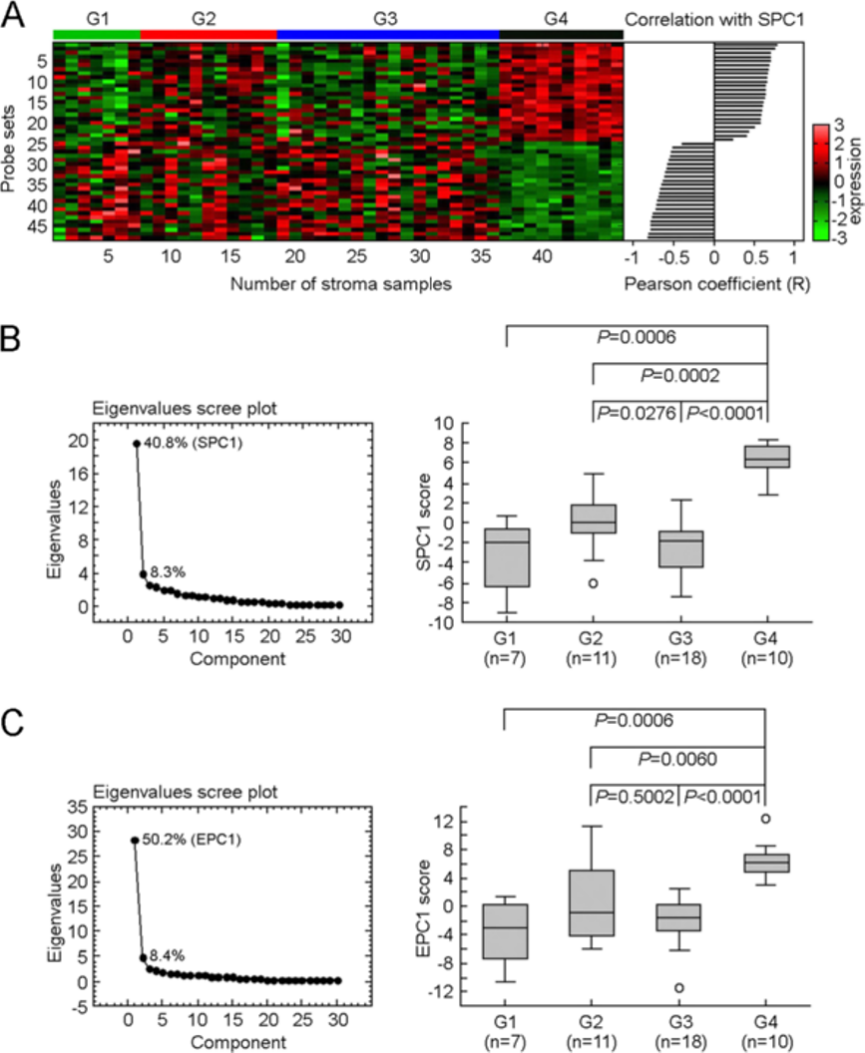
**Development of a stromal activation-associated 51-gene signature in tumor epithelium. (A)** Expression of 48 probe sets that were significantly up/downregulated in the stroma of subgroup G4 in 46 stromal samples. **(B)** Identify a principal component (SPC1) in stroma that represents collection group of 48 probe sets and measures differences of SPC1 score in stroma from different subgroups (n = 46). **(C)** Identify a principal component (EPC1) in tumor epithelium that represents the collection of stromal activation-associated genes (51 genes) and measures differences of EPC1 score in tumor epithelium from different subgroups (n = 46). Differences for each pair-wise comparison were assessed by Mann-Whitney *U* test. *Boxes* represent the 25% to 75% quartiles, *lines in the boxes* represent the median level, *whiskers* represent the non-outlier range, and *circles* represent the outliers.

Because reported experimental evidence suggests that the microenvironment of a malignant cell may play a critical role in breast cancer dormancy and late recurrences [40]-[44], we next sought to determine if the subgroup G4 was correlated with a microenvironment activation. We applied the subgroup classification to a subset of 46 paired samples [45], for which both tumor epithelium and stromal cell expression data were available. Using gene expression data of the tumor epithelium, we assigned these 46 samples into four subgroups and then compared gene expression profiles of their matched stromal cells. In this analysis, we found 48 probe sets whose expression was significantly up- or downregulated in the stroma of subgroup G4 samples, compared to samples in all other subgroup (false discovery rate (FDR) <0.05, Figure 5A, Additional file 5). However, there was no significant difference in expression of stromal cell genes among the other three subgroups (FDR <0.05, comparing subgroups G1, G2, and G3).

Owing to the heterogeneity of the cancer genome, individual genes might have only a modest effect on the phenotype, or account for only a fraction of the genetic basis of a phenotype; and as such, when several interactions occur together, the combined effect becomes robust and clinically significant [24],[46]. We therefore developed a principal component (SPC1, the first principal component of the 48 probe sets) comprising the entire set of genetic alterations identified in stromal of subgroup G4. Among subgroups with good prognosis (G3), early recurrence (G2) and late recurrence (G4), multiple group comparison revealed a linear correlation between the SPC1 score and time of recurrence (*P* = 1.05 × 10^−9^, ANOVA; Figure 5B), indicating this novel stromal activation in primary tumor might modulate delayed recurrence.

### Characteristics of the primary tumor and its microenvironment affect late distant metastasis

Although gene expression profiling has become the major tool for the study of breast cancer, a large collection of annotated tumor stromal data is not current available, which makes it difficult to validate stromal signatures. Therefore, we sought to find correlates of stromal activation in the expression analysis of malignant breast epithelial cells, and determined if tumor-induced changes in the stroma can serve as a surrogate for stromal activation, including the likelihood to escape dormancy. We performed a genome-scale linear regression analysis using the 46 paired tumors with both epithelium and stromal cell expression data, and identified 51 genes (57 probe sets) whose expressions were: (a) associated with SPC1 score (Pearson coefficient R >0.25); (b) specifically upregulated in subgroup G4 samples among 46 tumor epithelium samples (FDR <0.05, *t* test); and (c) directly correlated with higher risk of recurrence among 743 lymph node-negative breast tumor samples (*P* <0.01, Cox-regression survival analysis; Additional file 6). The first principal component (EPC1) that represents a collection of these 51 genes was significantly correlated with SPC1 score (Pearson coefficient R = 0.5952), and the distribution of EPC1 scores among different subgroups matched the pattern of SPC1 scores (Figure 5B and C), suggested the 51-gene signature in the tumor epithelium captured tumor-stroma interaction.

The top activated pathways of these 51 genes were ECM remodeling, fibrosis and EMT signaling (*TWIST1, JAG1, SNAI2*[30]), indicating the correlation between EMT and stromal activation in primary tumor with high risk of late recurrence (Additional file 4). Interestingly, we found a group of secreted proteins from the 51 genes that have been previously linked to distant metastasis (Additional file 6), including *POSTN*[47],[48], *TNC*[49]-[51], *VCAN*[52],[53], *MRC2*[54],[55], *ADAM9*[56],[57], *LIMS1*[58],[59] and *AEBP1*[60]. For instance, bone metastases from breast cancer induced by increased expression and serum secreted level of POSTN [47], and infiltrating tumor cells need to induce stromal POSTN expression in the secondary target organ to initiate colonization [48]. TNC expression has been correlated with higher risk of distant metastasis and local recurrence [49],[50], and breast cancer cells that infiltrate the lungs support their own metastasis-initiating ability by expressing TNC [51]. VCAN secretion is regulated by the primary tumor, and the level of VCAN deposited in the peritumoral stroma at the site of metastasis increased risk of breast cancer recurrence [52],[53]. Our results suggested that factors of the primary tumor might have a systemic effect on modulating both local and distant microenvironment, thereby influencing the fate of disseminated tumor cells (DTCs).

**Figure 6:**
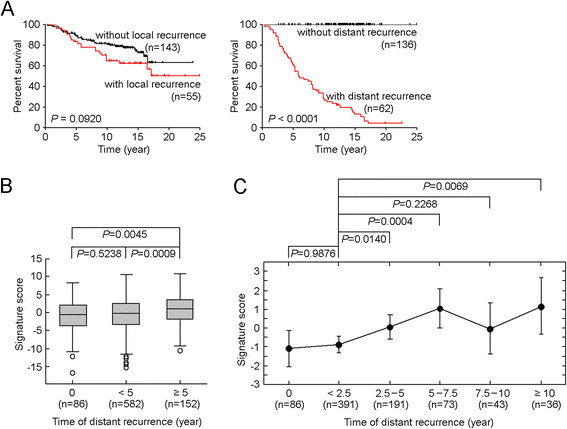
**The 51-gene signature was associated with late distant metastasis. (A)** Overall survival was stratified according to events of local or distant recurrence events among 198 lymph node-negative breast tumor samples obtained from patients who did not receive systemic neoadjuvant or adjuvant treatment. *Tick marks* in Kaplan-Meier estimates overall survival indicate patients whose data were censored by the time of last follow-up. *P* values were calculated using log-rank (Mantel-Cox) test. **(B)** Comparing the 51-gene signature score among patients with early or late distant metastasis in the 4,767 breast tumor dataset. ‘0’ represents samples that had no distant recurrence for more than 15 years. ‘<5’ and ‘≥5’ represent samples that had early or late distant metastasis. Pair-wise comparison was assessed using the exact Mann-Whitney *U* test. *Boxes* represent the 25% to 75% quartiles, *lines in the boxes* represent the median level, *whiskers* represent the non-outlier range, and *circles* represent the outliers. **(C)** Trend increase of the 51-gene signature score according to the time of distant metastasis. *Dots* represent average levels. *Vertical bars* represent 0.95 confidence intervals. Comparison of multiple groups was conducted using analysis of variance (ANOVA). Pair-wise comparison was assessed using the exact Mann-Whitney *U* test.

Death from breast cancer is most often due to metastatic disease rather than the primary tumor [61]. Among those 743 patients who did not received systemic neoadjuvant or adjuvant treatment, we found that breast cancer mortality was largely affected by distant metastasis or distant recurrence, while local recurrence did not significantly change the rate of overall survival (Figure 6A). Therefore, we next sought to determine the association of the 51-gene signature with distant metastasis/recurrence in a large independent cohort of patients, in which we had annotated distant metastasis data. Using the gene expression datasets of 4,767 breast cancer samples, we found that the 51-gene signature (EPC1, Additional file 7A) was significantly upregulated in primary tumors obtained from patients who had late distant metastasis, compared with samples with early distant metastasis (*P* = 0.0009, Mann-Whitney *U* test, Figure 6B). When samples were grouped according to time of distant metastasis, the 51-gene signature was significantly associated with time of distant recurrence (*P* = 0.0015, analysis of variance (ANOVA), Figure 6B), indicating tumor-driven stromal activation influences late disease recurrence in breast cancer independent of the molecular disease subtype.

### Concordance of epithelial-mesenchymal plasticity and stromal activation in primary tumors

Although only eight probe sets (14%) from the 51-gene signature overlapped with probe sets in gene cluster C4, we found that 25 of 27 probe sets (89.3%) from gene cluster C4 were significantly associated with stromal activation SPC1 score (Pearson coefficient R >0.25) among the 46 paired tumor epithelium and stroma samples. In an attempt to determine the correlation between the high degree of epithelial-mesenchymal plasticity of primary breast tumor (gene cluster C4) and the novel stromal activation (51-gene signature, EPC1), we developed a principal component (C4-PC1) that represents a collection of the 27 probe sets from gene cluster C4 among 4,767 breast cancer samples (Additional file 7B). We found that the C4-PC1 score was significantly associated with the 51-gene signature (Pearson coefficient R = 0.9303, Additional file 7C), and was significantly correlated with time of distant recurrence (*P* = 0.0011, ANOVA, Additional file 7D and E), indicating the concordance between high-degree epithelial-mesenchymal plasticity of tumor epithelium and stromal activation in the primary tumor.

It has been well recognized that the metastatic dissemination of cancer cells can occur in patients with early-stage cancer, even prior to initial clinical presentation [39],[62]; and this has also been seen in experimental models [63]-[65]. Yet, features of the primary tumor may not only control growth and the metastatic capacity of primary tumors, but also the ability of disseminated disease to shift into a state of dormancy [66]. While a key role of epithelial-mesenchymal plasticity in tumor dissemination has been well established in animal models [38],[39], the functional association of epithelial-mesenchymal plasticity in primary tumors with a delayed distant recurrence has not been shown in a large and well-characterized patient population. In fact, to our knowledge, there have been no reports on stromal changes in primary tumor that are predictive of late recurrence. Current study uncovered a disease subtype and tumor-stage independent gene signature in primary tumor epithelium that was associated with a novel stromal activation and a high degree of epithelial-mesenchymal plasticity of the primary tumor epithelium. The association with late recurrence suggested this 51-gene signature could predict the transition of tumor cells to a dormant phenotype with potential outgrowth as recurrent disease.

## Conclusions

In this study, we discovered a distinct set of genes that predicts late recurrence in breast cancer, and also show that early recurrence (recurrence within five years after initial treatment) was associated with upregulated stress response signaling and certain clinical parameters, such as molecular subtypes, tumor size and grade, while late recurrence (recurred ≥5 years after initial treatment) was associated with mesenchymal characteristics of the tumor epithelium and gene expression alterations in the adjacent tumor stroma. Though occurrence of late disease recurrence could be affected by genetic alterations acquired during the long latency of a dormant stage, the existence of a predictive gene signature for late recurrence in the primary tumor suggests that intrinsic features of this tumor govern the transition of disseminated tumor cells into a dormant phenotype with the ability to outgrowth as recurrent disease. Insight into these mechanisms could lead to the identification of novel biomarkers that indicate whether patients harbor dormant disease, and help uncover new signaling pathways that can be therapeutically manipulated to either eliminate dormant tumor cells or to indefinitely maintain them in this dormant state, thus preventing a progressive metastatic disease.

## Additional files

## Electronic supplementary material

Additional file 1: Summary of 25 data sets. Table summary of 4,767 samples obtained from 25 GEO data sets. (PDF 5 KB)

Additional file 2: **Heatmaps of data sets normalization.** **(A)** Heatmaps 4,767 expression data set. **(B)** Heatmaps of multi-tissue expression data set (n = 1,042). Heatmaps show the expression patterns in the data before and after normalization. The rows contain the 1,000 genes that exhibit the highest variance in gene expression profile across the original data set. The columns contain the samples in the data sets provided. The genes and samples are in the same order in both heatmaps. Warm colors indicate high expression of the gene, and cool colors indicate low expression. (PDF 194 KB)

Additional file 3: Early or late recurrence associated 216 probe sets. Table of 216 probe sets and their correlation with disease outcome. (PDF 48 KB)

Additional file 4: Significant pathways in selected gene sets. Table of top activated pathways of selected gene sets. (PDF 6 KB)

Additional file 5: Forty-eight probe sets that were up/downregulated in the stroma of subgroup G4. Table of 48 probe sets that were up/downregulated in the stroma of subgroup G4. (PDF 35 KB)

Additional file 6: Probe sets of 51-gene signature of stromal activation in primary tumor. Table of probe sets from 51-gene signature of stromal activation in primary tumor. (PDF 36 KB)

Additional file 7: **Correlation between gene cluster C4 and late distant metastasis in the 4,676 sample data set.** **(A)** Develop 51-gene signature (EPC1) in the 4,767 sample data set. **(B)** Develop principal component that represents gene cluster C4 in the 4,767 sample data set. **(C)** Pearson correlation between 51-gene signature (EPC1) and the first principal component of gene cluster C4 (C4-PC1) in the 4,767 sample data set. **(D)** Comparing the principal component of gene cluster C4 (C4-PC1) score among patients with early or late distant metastasis. Differences for each pair-wise comparison were assessed by Mann-Whitney *U* test. *Boxes* represent the 25% to 75% quartiles, *lines in the boxes* represent the median level, *whiskers* represent the non-outlier range, and *circles* represent the outliers. **(E)** Trend increasing of C4-PC1 score according to the time of distant metastasis. *Dots* represent average levels. *Vertical bars* represent 0.95 confidence intervals. Comparison of multiple groups was conducted using ANOVA. Pair-wise comparison was assessed using the exact Mann-Whitney *U* test. (PDF 226 KB)

Below are the links to the authors’ original submitted files for images.

Authors’ original file for figure 1

Authors’ original file for figure 2

Authors’ original file for figure 3

Authors’ original file for figure 4

Authors’ original file for figure 5

Authors’ original file for figure 6

Authors’ original file for figure 7

## Abbreviations

ANOVA: analysis of variance
BFRM: Bayesian Factor Regression Modeling
COXPH: Cox proportional-hazards regression survival analysis
DCIS: ductal carcinoma *in situ*
DTCs: disseminated tumor cells
ECM: extracellular matrix
EMT: epithelial to mesenchymal transition
ER: estrogen receptor
FDR: false discovery rate
GEO: Gene Expression Omnibus
GSEA: Gene Set Enrichment Analysis
HER2: human epidermal growth factor receptor 2
IHC: immunohistochemistry
PR: progesterone receptor
TGFβ: transforming growth factor beta
TNBC: triple-negative breast cancer

## References

[1] Uhr JW, Pantel K (2011). Controversies in clinical cancer dormancy. Proc Natl Acad Sci U S A.

[2] Fisher B, Jeong JH, Dignam J, Anderson S, Mamounas E, Wickerham DL, Wolmark N (2001). Findings from recent National Surgical Adjuvant Breast and Bowel Project adjuvant studies in stage I breast cancer. J Natl Cancer Inst Monogr.

[3] Wallgren A, Bonetti M, Gelber RD, Goldhirsch A, Castiglione-Gertsch M, Holmberg SB, Lindtner J, Thurlimann B, Fey M, Werner ID, Forbes JF, Price K, Coates AS, Collins J (2003). Risk factors for locoregional recurrence among breast cancer patients: results from International Breast Cancer Study Group Trials I through VII. J Clin Oncol.

[4] Aguirre-Ghiso JA (2007). Models, mechanisms and clinical evidence for cancer dormancy. Nat Rev Cancer.

[5] Demicheli R, Miceli R, Moliterni A, Zambetti M, Hrushesky WJ, Retsky MW, Valagussa P, Bonadonna G (2005). Breast cancer recurrence dynamics following adjuvant CMF is consistent with tumor dormancy and mastectomy-driven acceleration of the metastatic process. Ann Oncol.

[6] Wiedswang G, Borgen E, Karesen R, Qvist H, Janbu J, Kvalheim G, Nesland JM, Naume B (2004). Isolated tumor cells in bone marrow three years after diagnosis in disease-free breast cancer patients predict unfavorable clinical outcome. Clin Cancer Res.

[7] Braun S, Kentenich C, Janni W, Hepp F, De WJ, Willgeroth F, Sommer H, Pantel K (2000). Lack of effect of adjuvant chemotherapy on the elimination of single dormant tumor cells in bone marrow of high-risk breast cancer patients. J Clin Oncol.

[8] Becker S, Becker-Pergola G, Wallwiener D, Solomayer EF, Fehm T (2006). Detection of cytokeratin-positive cells in the bone marrow of breast cancer patients undergoing adjuvant therapy. Breast Cancer Res Treat.

[9] Staaf J, Ringner M, Vallon-Christersson J, Jonsson G, Bendahl PO, Holm K, Arason A, Gunnarsson H, Hegardt C, Agnarsson BA, Luts L, Grabau D, Ferno M, Malmstrom PO, Johannsson OT, Loman N, Barkardottir RB, Borg A (2010). Identification of subtypes in human epidermal growth factor receptor 2-positive breast cancer reveals a gene signature prognostic of outcome. J Clin Oncol.

[10] Brewster AM, Hortobagyi GN, Broglio KR, Kau SW, Santa-Maria CA, Arun B, Buzdar AU, Booser DJ, Valero V, Bondy M, Esteva FJ (2008). Residual risk of breast cancer recurrence 5 years after adjuvant therapy. J Natl Cancer Inst.

[11] Karrison TG, Ferguson DJ, Meier P (1999). Dormancy of mammary carcinoma after mastectomy. J Natl Cancer Inst.

[12] Demicheli R, Retsky MW, Swartzendruber DE, Bonadonna G (1997). Proposal for a new model of breast cancer metastatic development. Ann Oncol.

[13] Meltzer A (1990). Dormancy and breast cancer. J Surg Oncol.

[14] Chambers AF, Goss PE (2008). Putative growth characteristics of micrometastatic breast cancer. Breast Cancer Res.

[15] Demicheli R, Terenziani M, Bonadonna G (1998). Estimate of tumor growth time for breast cancer local recurrences: rapid growth after wake-up?. Breast Cancer Res Treat.

[16] Dao TL, Sunderland H (1959). Mammary carcinogenesis by 3-methylcholanthrene. I. Hormonal aspects in tumor induction and growth. J Natl Cancer Inst.

[17] Murray C (1995). Tumour dormancy: not so sleepy after all. Nat Med.

[18] Uhr JW, Scheuermann RH, Street NE, Vitetta ES (1997). Cancer dormancy: opportunities for new therapeutic approaches. Nat Med.

[19] Brackstone M, Townson JL, Chambers AF (2007). Tumour dormancy in breast cancer: an update. Breast Cancer Res.

[20] Willis L, Alarcon T, Elia G, Jones JL, Wright NA, Tomlinson IP, Graham TA, Page KM (2010). Breast cancer dormancy can be maintained by small numbers of micrometastases. Cancer Res.

[21] Cobleigh MA, Tabesh B, Bitterman P, Baker J, Cronin M, Liu ML, Borchik R, Mosquera JM, Walker MG, Shak S (2005). Tumor gene expression and prognosis in breast cancer patients with 10 or more positive lymph nodes. Clin Cancer Res.

[22] Parker JS, Mullins M, Cheang MC, Leung S, Voduc D, Vickery T, Davies S, Fauron C, He X, Hu Z, Quackenbush JF, Stijleman IJ, Palazzo J, Marron JS, Nobel AB, Mardis E, Nielsen TO, Ellis MJ, Perou CM, Bernard PS (2009). Supervised risk predictor of breast cancer based on intrinsic subtypes. J Clin Oncol.

[23] Van DV, He YD, Van’t Veer LJ, Dai H, Hart AA, Voskuil DW, Schreiber GJ, Peterse JL, Roberts C, Marton MJ, Parrish M, Atsma D, Witteveen A, Glas A, Delahaye L, Van DV, Bartelink H, Rodenhuis S, Rutgers ET, Friend SH, Bernards R (2002). A gene-expression signature as a predictor of survival in breast cancer. N Engl J Med.

[24] Cheng Q, Chang JT, Geradts J, Neckers LM, Haystead T, Spector N, Lyerly HK (2012). Amplification and high-level expression of heat shock protein 90 marks aggressive phenotypes of human epidermal growth factor receptor 2 negative breast cancer. Breast Cancer Res.

[25] Chang JT, Gatza ML, Lucas JE, Barry WT, Vaughn P, Nevins JR (2011). SIGNATURE: a workbench for gene expression signature analysis. BMC Bioinformatics.

[26] Subramanian A, Tamayo P, Mootha VK, Mukherjee S, Ebert BL, Gillette MA, Paulovich A, Pomeroy SL, Golub TR, Lander ES, Mesirov JP (2005). Gene set enrichment analysis: a knowledge-based approach for interpreting genome-wide expression profiles. Proc Natl Acad Sci U S A.

[27] Mootha VK, Lindgren CM, Eriksson KF, Subramanian A, Sihag S, Lehar J, Puigserver P, Carlsson E, Ridderstrale M, Laurila E, Houstis N, Daly MJ, Patterson N, Mesirov JP, Golub TR, Tamayo P, Spiegelman B, Lander ES, Hirschhorn JN, Altshuler D, Groop LC (2003). PGC-1alpha-responsive genes involved in oxidative phosphorylation are coordinately downregulated in human diabetes. Nat Genet.

[28] Savagner P (2010). The epithelial-mesenchymal transition (EMT) phenomenon. Ann Oncol.

[29] Yang J, Mani SA, Donaher JL, Ramaswamy S, Itzykson RA, Come C, Savagner P, Gitelman I, Richardson A, Weinberg RA (2004). Twist, a master regulator of morphogenesis, plays an essential role in tumor metastasis. Cell.

[30] De CB, Berx G (2013). Regulatory networks defining EMT during cancer initiation and progression. Nat Rev Cancer.

[31] Yang LT, Nichols JT, Yao C, Manilay JO, Robey EA, Weinmaster G (2005). Fringe glycosyltransferases differentially modulate Notch1 proteolysis induced by Delta1 and Jagged1. Mol Biol Cell.

[32] Pessah M, Prunier C, Marais J, Ferrand N, Mazars A, Lallemand F, Gauthier JM, Atfi A (2001). c-Jun interacts with the corepressor TG-interacting factor (TGIF) to suppress Smad2 transcriptional activity. Proc Natl Acad Sci U S A.

[33] Wotton D, Lo RS, Lee S, Massague J (1999). A Smad transcriptional corepressor. Cell.

[34] Stroschein SL, Wang W, Zhou S, Zhou Q, Luo K (1999). Negative feedback regulation of TGF-beta signaling by the SnoN oncoprotein. Science.

[35] Colland F, Jacq X, Trouplin V, Mougin C, Groizeleau C, Hamburger A, Meil A, Wojcik J, Legrain P, Gauthier JM (2004). Functional proteomics mapping of a human signaling pathway. Genome Res.

[36] Brabletz T, Jung A, Reu S, Porzner M, Hlubek F, Kunz-Schughart LA, Knuechel R, Kirchner T (2001). Variable beta-catenin expression in colorectal cancers indicates tumor progression driven by the tumor environment. Proc Natl Acad Sci U S A.

[37] Mejlvang J, Kriajevska M, Vandewalle C, Chernova T, Sayan AE, Berx G, Mellon JK, Tulchinsky E (2007). Direct repression of cyclin D1 by SIP1 attenuates cell cycle progression in cells undergoing an epithelial mesenchymal transition. Mol Biol Cell.

[38] Brabletz T (2012). To differentiate or not–routes towards metastasis. Nat Rev Cancer.

[39] Kang Y, Pantel K (2013). Tumor cell dissemination: emerging biological insights from animal models and cancer patients. Cancer Cell.

[40] Dvorak HF (1986). Tumors: wounds that do not heal. Similarities between tumor stroma generation and wound healing. N Engl J Med.

[41] Wiseman BS, Werb Z (2002). Stromal effects on mammary gland development and breast cancer. Science.

[42] Mueller MM, Fusenig NE (2004). Friends or foes - bipolar effects of the tumour stroma in cancer. Nat Rev Cancer.

[43] Joyce JA, Pollard JW (2009). Microenvironmental regulation of metastasis. Nat Rev Cancer.

[44] Bissell MJ, Hines WC (2011). Why don’t we get more cancer? A proposed role of the microenvironment in restraining cancer progression. Nat Med.

[45] Boersma BJ, Reimers M, Yi M, Ludwig JA, Luke BT, Stephens RM, Yfantis HG, Lee DH, Weinstein JN, Ambs S (2008). A stromal gene signature associated with inflammatory breast cancer. Int J Cancer.

[46] Diouf B, Cheng Q, Krynetskaia NF, Yang W, Cheok M, Pei D, Fan Y, Cheng C, Krynetskiy EY, Geng H, Chen S, Thierfelder WE, Mullighan CG, Downing JR, Hsieh P, Pui CH, Relling MV, Evans WE (2011). Somatic deletions of genes regulating MSH2 protein stability cause DNA mismatch repair deficiency and drug resistance in human leukemia cells. Nat Med.

[47] Contie S, Voorzanger-Rousselot N, Litvin J, Clezardin P, Garnero P (2011). Increased expression and serum levels of the stromal cell-secreted protein periostin in breast cancer bone metastases. Int J Cancer.

[48] Malanchi I, Santamaria-Martinez A, Susanto E, Peng H, Lehr HA, Delaloye JF, Huelsken J (2012). Interactions between cancer stem cells and their niche govern metastatic colonization. Nature.

[49] Jahkola T, Toivonen T, Virtanen I, Von SK, Nordling S, Von BK, Haglund C, Nevanlinna H, Blomqvist C (1998). Tenascin-C expression in invasion border of early breast cancer: a predictor of local and distant recurrence. Br J Cancer.

[50] Tsunoda T, Inada H, Kalembeyi I, Imanaka-Yoshida K, Sakakibara M, Okada R, Katsuta K, Sakakura T, Majima Y, Yoshida T (2003). Involvement of large tenascin-C splice variants in breast cancer progression. Am J Pathol.

[51] Oskarsson T, Acharyya S, Zhang XH, Vanharanta S, Tavazoie SF, Morris PG, Downey RJ, Manova-Todorova K, Brogi E, Massague J (2011). Breast cancer cells produce tenascin C as a metastatic niche component to colonize the lungs. Nat Med.

[52] Ricciardelli C, Brooks JH, Suwiwat S, Sakko AJ, Mayne K, Raymond WA, Seshadri R, LeBaron RG, Horsfall DJ (2002). Regulation of stromal versican expression by breast cancer cells and importance to relapse-free survival in patients with node-negative primary breast cancer. Clin Cancer Res.

[53] Brown LF, Guidi AJ, Schnitt SJ, Van De WL, Iruela-Arispe ML, Yeo TK, Tognazzi K, Dvorak HF (1999). Vascular stroma formation in carcinoma in situ, invasive carcinoma, and metastatic carcinoma of the breast. Clin Cancer Res.

[54] Curino AC, Engelholm LH, Yamada SS, Holmbeck K, Lund LR, Molinolo AA, Behrendt N, Nielsen BS, Bugge TH (2005). Intracellular collagen degradation mediated by uPARAP/Endo180 is a major pathway of extracellular matrix turnover during malignancy. J Cell Biol.

[55] Wienke D, Davies GC, Johnson DA, Sturge J, Lambros MB, Savage K, Elsheikh SE, Green AR, Ellis IO, Robertson D, Reis-Filho JS, Isacke CM (2007). The collagen receptor Endo180 (CD280) Is expressed on basal-like breast tumor cells and promotes tumor growth in vivo. Cancer Res.

[56] Mazzocca A, Coppari R, De FR, Cho JY, Libermann TA, Pinzani M, Toker A (2005). A secreted form of ADAM9 promotes carcinoma invasion through tumor-stromal interactions. Cancer Res.

[57] O’Shea C, McKie N, Buggy Y, Duggan C, Hill AD, McDermott E, O’Higgins N, Duffy MJ (2003). Expression of ADAM-9 mRNA and protein in human breast cancer. Int J Cancer.

[58] Wang-Rodriguez J, Dreilinger AD, Alsharabi GM, Rearden A (2002). The signaling adapter protein PINCH is up-regulated in the stroma of common cancers, notably at invasive edges. Cancer.

[59] Scaife CL, Shea J, Emerson L, Boucher K, Firpo MA, Beckerle MC, Mulvihill SJ (2010). Prognostic significance of PINCH signalling in human pancreatic ductal adenocarcinoma. HPB (Oxford).

[60] Holloway RW, Bogachev O, Bharadwaj AG, McCluskey GD, Majdalawieh AF, Zhang L, Ro HS (2012). Stromal adipocyte enhancer-binding protein (AEBP1) promotes mammary epithelial cell hyperplasia via proinflammatory and hedgehog signaling. J Biol Chem.

[61] Jemal A, Siegel R, Ward E, Hao Y, Xu J, Murray T, Thun MJ (2008). Cancer statistics, 2008. CA Cancer J Clin.

[62] Pantel K, Brakenhoff RH (2004). Dissecting the metastatic cascade. Nat Rev Cancer.

[63] Husemann Y, Geigl JB, Schubert F, Musiani P, Meyer M, Burghart E, Forni G, Eils R, Fehm T, Riethmuller G, Klein CA (2008). Systemic spread is an early step in breast cancer. Cancer Cell.

[64] Eyles J, Puaux AL, Wang X, Toh B, Prakash C, Hong M, Tan TG, Zheng L, Ong LC, Jin Y, Kato M, Prevost-Blondel A, Chow P, Yang H, Abastado JP (2010). Tumor cells disseminate early, but immunosurveillance limits metastatic outgrowth, in a mouse model of melanoma. J Clin Invest.

[65] Riethmuller G, Klein CA (2001). Early cancer cell dissemination and late metastatic relapse: clinical reflections and biological approaches to the dormancy problem in patients. Semin Cancer Biol.

[66] Wang Y, Klijn JG, Zhang Y, Sieuwerts AM, Look MP, Yang F, Talantov D, Timmermans M, Meijer-van Gelder ME, Yu J, Jatkoe T, Berns EM, Atkins D, Foekens JA (2005). Gene-expression profiles to predict distant metastasis of lymph-node-negative primary breast cancer. Lancet.

