# Supplementary material for: A signature of epithelial-mesenchymal plasticity and stromal activation in primary tumor modulates late recurrence in breast cancer independent of disease subtype
Source: Breast Cancer Res. 2014 Jul 25;16:407. doi: 10.1186/s13058-014-0407-9 (PMC4187325; doi:10.1186/s13058-014-0407-9)
Supplement: Supplementary file 1 — Additional file 1: Summary of 25 data sets. Table summary of 4,767 samples obtained from 25 GEO data sets. (PDF 5 KB) [file 13058_2014_407_MOESM1_ESM.pdf]

Additional file 1 . Summary of 25 datasets

Data set	Platform	No. of array	prognosis	clinical parameters
GSE11121	GPL96	200	dmfs	Nodal, Grade, Size
GSE12093	GPL96	136	dmfs (distant relapse)	ER, Nodal
GSE12276	GPL570	204	rfs (local, distant)	ER
GSE1456	GPL96	159	rfs, os, Death from BC	Grade
GSE16391	GPL570	55	rfs	HER2, ER, PR, Size, Age, Grade, Nodal
GSE16446	GPL570	120	os,dmfs	HER2, ER, PR, Tstg, Nstg, Age, Grade
GSE17705	GPL96	298	dmfs (distant relapse)	ER, Nodal
GSE17907	GPL570	55	dmfs	HER2, ER, PR, Grade, Age, His
GSE19615	GPL570	115	dmfs (distant relapse)	HER2, ER, PR, Age, Size, Nodal
GSE2034	GPL96	286	rfs	ER, Nodal
GSE20685	GPL570	327	os, dmfs	Age
GSE21653	GPL570	266	rfs (disease free)	HER2, ER, PR, Age, Grade, Tstg, P53
GSE22035	GPL570	43		ER, Age
GSE22093	GPL96	82		HER2, ER, Age, Tstg, Size, Nodal, Grade
GSE23177	GPL570	116		ER, HER2, Grade, His
GSE23720	GPL570	197		ER, PR, Age
GSE25066	GPL96	508	dmfs (distant relapse)	HER2, ER, PR, Age, Tstg, Stage, Nodal, Grade
GSE26639	GPL570	226		HER2, ER, PR, Grade
GSE3494	GPL96	251	Death from BC	ER, PR, Grade, Nodal, Size, Age, P53
GSE4922	GPL96	249	rfs (disease free)	ER, Grade, Size, Age, P53
GSE5327	GPL96	58	dmfs	ER
GSE5460	GPL570	129		HER2, ER, Grade, Size, His
GSE6532	GPL96/570	414	rfs, dmfs	ER, PR, Age, Nodal, Size, Grade
GSE7390	GPL96	198	os, rfs, dmfs	ER, Nodal, Size, His, Grade
GSE9195	GPL570	77	rfs, dmfs	ER, PR, Nodal, Size, Age

* GPL96, Affymetrix U133A; GPL570, Affymetrix U133 Plus 2.0; os, over-all survival; rfs, recurrence-free survival; dmfs, distant metastasis-free survival; Tstg, Tumor stage; Nstg, Nodal stage; His, histology information;
