# Supplementary figures and images for: A signature of epithelial-mesenchymal plasticity and stromal activation in primary tumor modulates late recurrence in breast cancer independent of disease subtype
Source: Breast Cancer Res. 2014 Jul 25;16:407. doi: 10.1186/s13058-014-0407-9 (PMC4187325; doi:10.1186/s13058-014-0407-9)

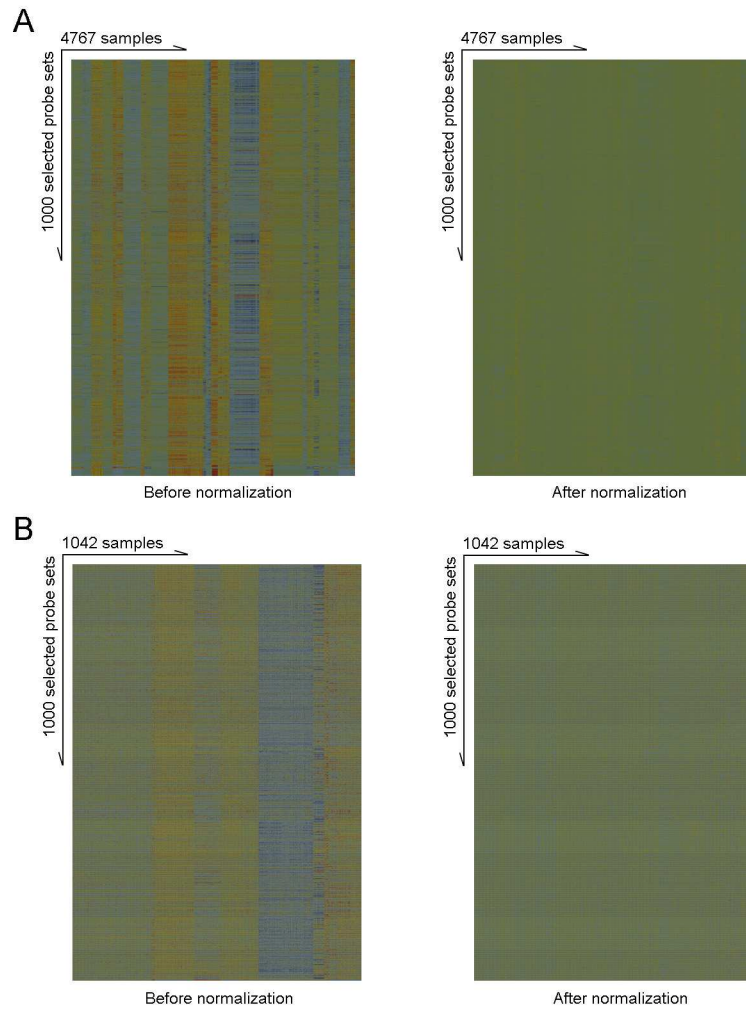

Supplement: Supplementary file 2 — Additional file 2: Heatmaps of data sets normalization. (A) Heatmaps 4,767 expression data set. (B) Heatmaps of multi-tissue expression data set (n = 1,042). Heatmaps show the expression patterns in the data before and after normalization. The rows contain the 1,000 genes that exhibit the highest variance in gene expression profile across the original data set. The columns contain the samples in the data sets provided. The genes and samples are in the same order in both heatmaps. Warm colors indicate high expression of the gene, and cool colors indicate low expression. (PDF 194 KB) [file 13058_2014_407_MOESM2_ESM.pdf]

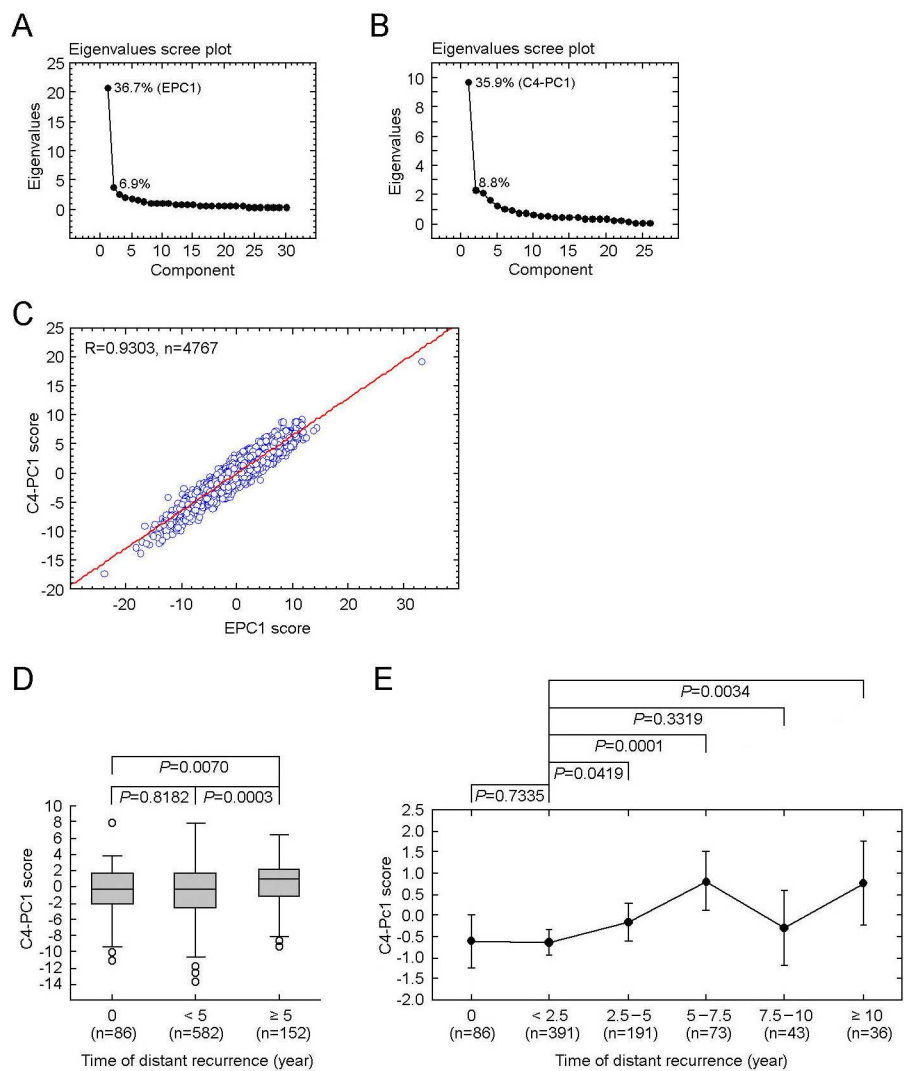

Supplement: Supplementary file 7 — Additional file 7: Correlation between gene cluster C4 and late distant metastasis in the 4,676 sample data set. (A) Develop 51-gene signature (EPC1) in the 4,767 sample data set. (B) Develop principal component that represents gene cluster C4 in the 4,767 sample data set. (C) Pearson correlation between 51-gene signature (EPC1) and the first principal component of gene cluster C4 (C4-PC1) in the 4,767 sample data set. (D) Comparing the principal component of gene cluster C4 (C4-PC1) score among patients with early or late distant metastasis. Differences for each pair-wise comparison were assessed by Mann-Whitney U test. Boxes represent the 25% to 75% quartiles, lines in the boxes represent the median level, whiskers represent the non-outlier range, and circles represent the outliers. (E) Trend increasing of C4-PC1 score according to the time of distant metastasis. Dots represent average levels. Vertical bars represent 0.95 confidence intervals. Comparison of multiple groups was conducted using ANOVA. Pair-wise comparison was assessed using the exact Mann-Whitney U test. (PDF 226 KB) [file 13058_2014_407_MOESM7_ESM.pdf]

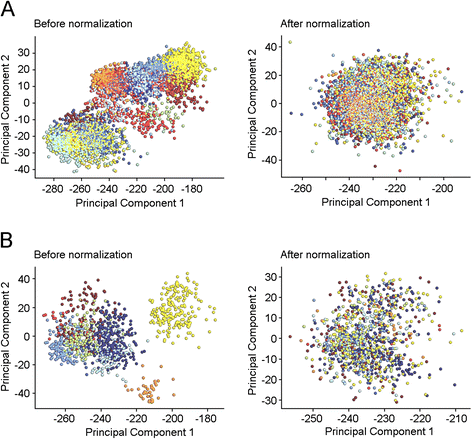

Supplement: Supplementary file 8 — Authors’ original file for figure 1 [file 13058_2014_407_MOESM8_ESM.gif]

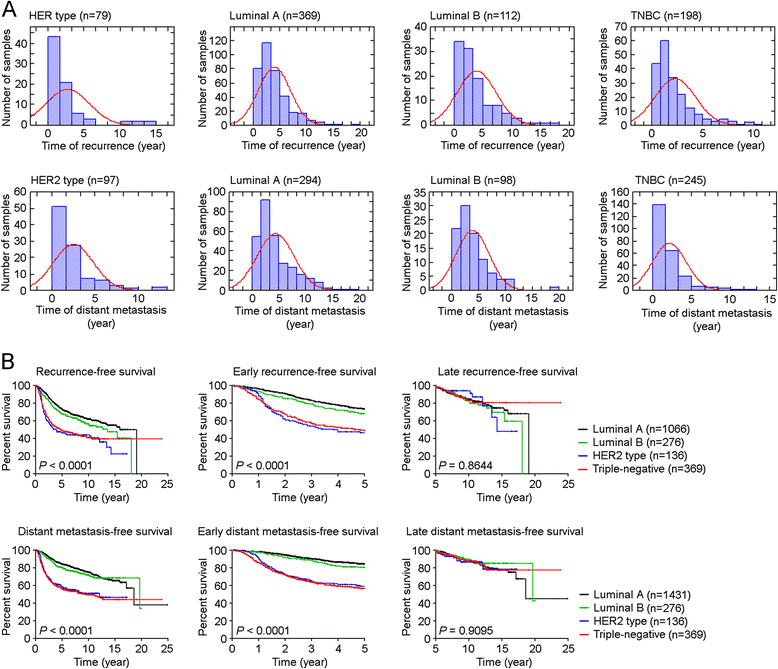

Supplement: Supplementary file 9 — Authors’ original file for figure 2 [file 13058_2014_407_MOESM9_ESM.gif]

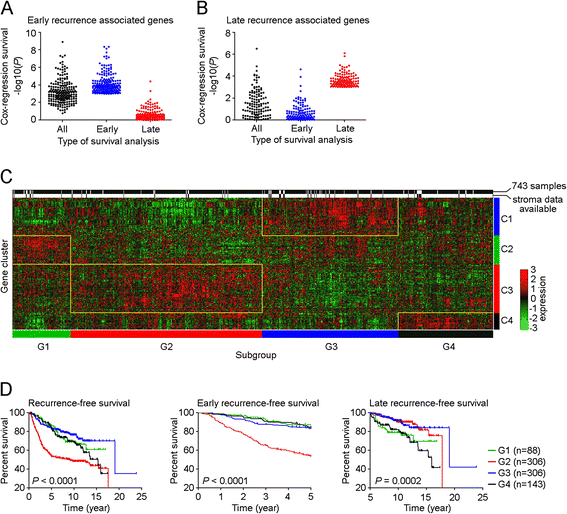

Supplement: Supplementary file 10 — Authors’ original file for figure 3 [file 13058_2014_407_MOESM10_ESM.gif]

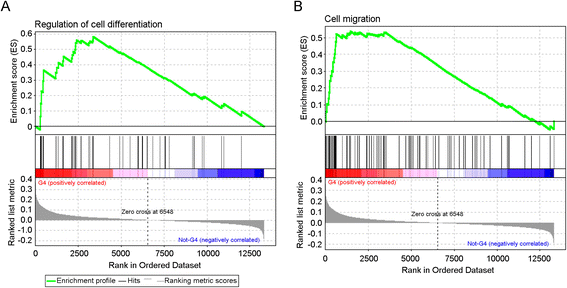

Supplement: Supplementary file 11 — Authors’ original file for figure 4 [file 13058_2014_407_MOESM11_ESM.gif]

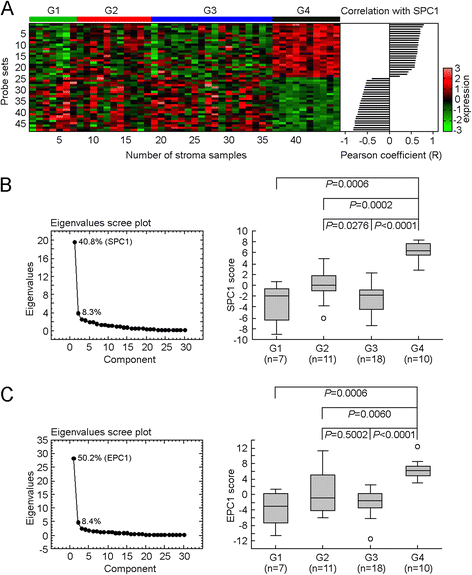

Supplement: Supplementary file 12 — Authors’ original file for figure 5 [file 13058_2014_407_MOESM12_ESM.gif]

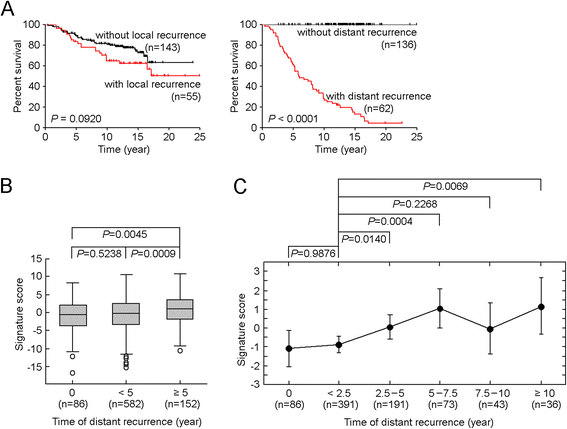

Supplement: Supplementary file 13 — Authors’ original file for figure 6 [file 13058_2014_407_MOESM13_ESM.gif]
