# Supplementary material for: A signature of epithelial-mesenchymal plasticity and stromal activation in primary tumor modulates late recurrence in breast cancer independent of disease subtype
Source: Breast Cancer Res. 2014 Jul 25;16:407. doi: 10.1186/s13058-014-0407-9 (PMC4187325; doi:10.1186/s13058-014-0407-9)
Supplement: Supplementary file 3 — Additional file 3: Early or late recurrence associated 216 probe sets. Table of 216 probe sets and their correlation with disease outcome. (PDF 48 KB) [file 13058_2014_407_MOESM3_ESM.pdf]

Additional file 3. Early or late recurrence associated 216 probe sets

NAME	Gene symbol	Cluster	Early recurrence-free survival				Late recurrence-free survival			
			Cox-regression		COXPH		Cox-regression		COXPH	
			p-value	coeff.	p-value	coeff.	p-value	coeff.	p-value	coeff.
201010_s_at	TXNIP	C1	4.64E-06	-0.5602	0.0010	-0.6595	0.3109	0.2204	0.1264	0.4117
204959_at	MNDA	C1	5.58E-04	-0.3726	0.0054	-0.4576	0.7635	0.0508	0.8113	-0.0515
209354_at	TNFRSF14	C1	4.81E-05	-0.7832	0.0018	-0.9308	0.2472	0.3625	0.1953	0.4871
202216_x_at	NFYC	C1	1.59E-06	-1.0928	0.0095	-0.7764	0.1537	0.4975	0.0205	0.9727
218322_s_at	ACSL5	C1	2.53E-04	-0.5762	7.86E-04	-0.7080	0.7263	-0.0809	0.9704	-0.0106
200918_s_at	SRPR	C1	2.65E-04	-0.6475	0.0021	-0.7444	0.5291	-0.1748	0.7394	0.1075
218774_at	DCPS	C1	4.19E-04	-0.6865	8.56E-04	-0.8264	0.0502	-0.6127	0.5358	-0.2169
206781_at	DNAJC4	C1	1.04E-04	-1.1330	0.0067	-1.2352	0.6447	-0.2245	0.9212	-0.0626
201288_at	ARHGDIB	C1	3.20E-05	-0.4945	8.95E-04	-0.5881	0.6537	0.0926	0.5796	0.1451
200901_s_at	M6PR	C1	6.63E-04	-0.6823	0.0025	-0.8432	0.0102	-0.8539	0.0545	-0.8182
218746_at	TAPBPL	C1	1.52E-06	-0.7778	8.91E-05	-0.8827	0.2183	0.2881	0.1512	0.3946
218747_s_at	TAPBPL	C1	1.09E-06	-0.7245	2.42E-05	-0.9241	0.9795	0.0057	0.4129	0.2172
208871_at	ATN1	C1	4.69E-04	-0.8017	0.0073	-0.8965	0.8545	-0.0651	0.4321	-0.3463
209374_s_at	IGHM	C1	6.03E-05	-0.1984	2.49E-04	-0.2483	0.7637	0.0242	0.0310	0.2255
205101_at	CIITA	C1	2.16E-05	-1.0504	0.0024	-0.9956	0.0615	0.6128	0.0016	1.2798
209083_at	CORO1A	C1	2.58E-04	-0.4284	0.0014	-0.5127	0.3526	0.1685	0.1386	0.3283
204683_at	ICAM2	C1	8.90E-04	-0.5801	0.0031	-0.6612	0.9166	-0.0245	0.9012	0.0327
219218_at	BAHCC1	C1	3.27E-04	-0.7459	0.0074	-0.8216	0.2238	-0.3827	0.0433	-0.8719
205926_at	IL27RA	C1	4.16E-05	-0.7389	6.33E-04	-0.9214	0.0111	-0.7506	0.4317	-0.2671
209152_s_at	TCF3	C1	0.2104	-0.2857	0.4239	-0.2533	1.06E-04	-1.4597	0.0069	-1.2134
213915_at	NKG7	C1	3.47E-04	-0.4982	0.0038	-0.4915	0.6106	0.1003	0.1954	0.2891
206881_s_at	LILRA3	C1	7.74E-05	-1.8079	0.0084	-1.7186	0.8934	-0.1089	0.9976	0.0030
201368_at	ZFP36L2	C1	8.06E-06	-0.4868	0.0068	-0.4619	0.7686	0.0560	0.8894	-0.0337
214669_x_at	IGKC	C1	2.33E-04	-0.1954	0.0019	-0.2394	0.5334	-0.0584	0.2486	0.1501
214836_x_at	IGKC	C1	5.37E-04	-0.2263	0.0028	-0.2730	0.4545	-0.0827	0.5001	0.0985
201642_at	IFNGR2	C1	1.66E-04	-0.8089	0.0037	-0.8678	0.1041	-0.5556	0.0193	-1.0743
215214_at	IGL@	C1	6.14E-04	-0.2617	0.0016	-0.2973	0.5758	-0.0562	0.9675	-0.0045
209138_x_at	IGL@	C1	7.18E-04	-0.1451	9.44E-04	-0.2078	0.4859	-0.0533	0.3074	0.1104
217148_x_at	IGL@	C1	1.43E-05	-0.2819	1.57E-05	-0.3496	0.5091	-0.0618	0.8222	0.0245
215946_x_at	IGLL3	C1	5.38E-04	-0.2674	0.0019	-0.3223	0.4344	-0.0996	0.3424	0.1548
221087_s_at	APOL3	C1	1.82E-06	-0.9097	1.46E-04	-0.9777	0.5861	0.1720	0.1151	0.6078
220005_at	P2RY13	C1	9.37E-04	-0.5681	0.0017	-0.7687	0.8635	0.0434	0.9916	-0.0031
212646_at	RFTN1	C1	3.67E-04	-0.4289	7.33E-04	-0.5546	0.6454	0.0770	0.5651	0.1119
202531_at	IRF1	C1	6.52E-06	-0.8226	3.62E-04	-0.8254	0.9827	0.0061	0.9328	0.0278
209619_at	CD74	C1	3.14E-09	-0.7176	5.57E-05	-0.6885	0.0019	0.7022	0.0143	0.6804
210285_x_at	WTAP	C1	4.71E-05	-0.6282	0.0049	-0.5719	0.5065	-0.1660	0.2220	-0.3575
217984_at	RNASET2	C1	9.84E-06	-0.6442	0.0017	-0.6491	0.7211	0.0877	0.3262	0.3017
217983_s_at	RNASET2	C1	7.03E-05	-0.4940	9.78E-04	-0.5942	0.5302	0.1307	0.4509	0.2045
205298_s_at	BTN2A2	C1	3.05E-04	-0.7335	0.0047	-0.7359	0.6654	-0.1399	0.7041	0.1443
204806_x_at	HLA-F	C1	9.47E-06	-0.6572	0.0042	-0.5530	0.0721	0.4591	0.0044	0.8260

Additional file 3. Early or late recurrence associated 216 probe sets (continued)

NAME	Gene symbol	Cluster	Early recurrence-free survival				Late recurrence-free survival			
			Cox-regression		COXPH		Cox-regression		COXPH	
			p-value	coeff.	p-value	coeff.	p-value	coeff.	p-value	coeff.
210514_x_at	HLA-G	C1	1.50E-04	-0.6761	0.0095	-0.6021	0.1608	0.4414	0.0319	0.7604
211529_x_at	HLA-G	C1	1.96E-05	-0.6722	8.93E-04	-0.6476	0.0168	0.6997	0.0039	0.9195
211528_x_at	HLA-G	C1	5.56E-05	-0.6794	0.0018	-0.6597	0.0511	0.5555	0.0308	0.6715
208729_x_at	HLA-B	C1	1.96E-04	-0.4551	0.0044	-0.4536	0.0258	0.4845	0.0012	0.8116
208812_x_at	HLA-C	C1	1.14E-04	-0.7363	7.89E-04	-0.8189	0.1596	0.4548	0.0610	0.6666
214459_x_at	HLA-C	C1	3.32E-05	-0.7871	4.98E-04	-0.8529	0.1268	0.4930	0.0149	0.8762
211911_x_at	HLA-B	C1	7.29E-06	-0.5583	4.62E-04	-0.5672	0.0177	0.5108	0.0054	0.6789
208894_at	HLA-DRA	C1	7.77E-07	-0.4385	7.48E-05	-0.5187	0.0058	0.4425	0.0244	0.4285
210982_s_at	HLA-DRA	C1	2.75E-06	-0.4295	2.25E-05	-0.5673	0.0154	0.3909	0.0377	0.3979
217362_x_at	HLA-DRB6	C1	1.89E-06	-0.8638	4.37E-04	-0.8757	0.6724	0.1298	0.9682	0.0153
211656_x_at	HLA-DQB1	C1	2.11E-04	-0.4150	0.0080	-0.3985	0.1940	0.2415	0.3409	0.2030
211654_x_at	HLA-DQB1	C1	4.65E-04	-0.2964	1.03E-04	-0.4319	0.0573	0.2517	0.0101	0.4000
205671_s_at	HLA-DOB	C1	5.17E-04	-0.5310	0.0042	-0.5856	0.0784	-0.4076	0.4064	-0.2319
217478_s_at	HLA-DMA	C1	1.67E-07	-0.5196	3.77E-05	-0.6053	0.0960	0.2999	0.0466	0.4368
211991_s_at	HLA-DPA1	C1	8.53E-07	-0.4312	3.24E-05	-0.5183	0.0378	0.3141	0.0495	0.3556
211990_at	HLA-DPA1	C1	1.43E-04	-0.3538	5.35E-05	-0.5135	0.1125	0.3000	0.2504	0.2624
213537_at	HLA-DPA1	C1	8.18E-05	-0.3765	1.57E-05	-0.5281	0.2620	0.1785	0.2230	0.2301
201137_s_at	HLA-DPB1	C1	2.41E-05	-0.4163	0.0041	-0.3969	4.15E-04	0.5919	0.0017	0.6292
208829_at	TAPBP	C1	4.07E-04	-0.5605	0.0090	-0.5329	0.7453	-0.0776	0.6652	0.1202
209034_at	PNRC1	C1	3.06E-04	-0.6371	0.0078	-0.6407	0.0082	-0.7304	0.0634	-0.6299
202111_at	SLC4A2	C1	0.6103	-0.1014	0.1907	-0.3770	1.10E-04	1.1430	4.80E-04	1.2418
204834_at	FGL2	C1	4.85E-05	-0.5175	2.41E-04	-0.6644	0.7979	0.0516	0.6020	0.1209
203528_at	SEMA4D	C1	4.65E-04	-0.5901	5.39E-05	-0.9543	0.7051	-0.1048	0.5374	0.2147
202337_at	PMF1	C2	4.82E-04	-0.6867	7.17E-04	-0.9002	0.9633	-0.0137	0.8718	-0.0574
202709_at	FMOD	C2	2.11E-04	-0.3878	0.0037	-0.4236	0.4237	0.1139	0.9802	0.0041
221435_x_at	HYI	C2	8.06E-06	-1.1804	0.0022	-1.0859	0.2594	0.3912	0.2520	0.4519
209472_at	CCBL2	C2	5.53E-04	-0.4470	0.0020	-0.5432	0.0424	-0.4144	0.0057	-0.6901
211376_s_at	NSMCE4A	C2	6.63E-04	-0.5520	0.0024	-0.6867	0.1881	-0.3441	0.1042	-0.4961
219469_at	DYNC2H1	C2	4.67E-04	-0.5309	0.0038	-0.6380	0.6130	-0.1130	0.1518	-0.4018
203509_at	SORL1	C2	5.37E-04	-0.4051	0.0061	-0.4195	0.9412	0.0126	0.3166	-0.1939
212560_at	SORL1	C2	8.05E-04	-0.3049	9.39E-05	-0.4944	0.6193	0.0748	0.3885	-0.1509
218491_s_at	THYN1	C2	1.78E-05	-0.6334	0.0079	-0.5403	0.2686	0.2709	0.6170	0.1483
49878_at	PEX16	C2	0.8203	-0.0878	0.6859	0.2021	1.47E-05	2.4169	2.78E-04	2.6568
202117_at	ARHGAP1	C2	7.15E-04	0.7422	0.0047	0.8681	0.0259	0.8169	0.2121	0.5510
211779_x_at	AP2A2	C2	7.46E-05	-0.8654	0.0084	-0.8276	0.0417	0.7684	0.0026	1.4543
209075_s_at	ISCU	C2	6.63E-04	-0.6120	0.0041	-0.8456	0.1350	0.4573	0.3543	0.3689
200794_x_at	DAZAP2	C2	0.8294	-0.0436	0.6747	-0.1193	1.11E-04	1.4350	0.0032	1.3503
214334_x_at	DAZAP2	C2	0.1502	0.2628	0.3604	0.2721	7.06E-05	1.3028	4.27E-04	1.5742
219956_at	GALNT6	C2	0.4666	0.0423	0.0163	0.2010	7.18E-05	0.4011	9.15E-04	0.4170
205399_at	DCLK1	C2	0.3644	-0.0563	0.1818	-0.1258	5.07E-05	0.3248	4.52E-06	0.4677

Additional file 3. Early or late recurrence associated 216 probe sets (continued)

NAME	Gene symbol	Cluster	Early recurrence-free survival				Late recurrence-free survival			
			Cox-regression		COXPH		Cox-regression		COXPH	
			p-value	coeff.	p-value	coeff.	p-value	coeff.	p-value	coeff.
203485_at	RTN1	C2	4.13E-05	-0.2991	8.25E-05	-0.4182	0.2479	0.1026	0.9633	-0.0048
210222_s_at	RTN1	C2	1.07E-05	-0.3770	1.55E-04	-0.4608	0.9455	-0.0071	0.2770	-0.1327
212600_s_at	UQCRC2	C2	3.23E-04	-0.5895	0.0017	-0.7320	0.5639	0.1525	0.7813	-0.0893
221666_s_at	PYCARD	C2	1.02E-04	-0.2853	0.0077	-0.2854	0.9795	-0.0031	0.6756	-0.0616
201508_at	IGFBP4	C2	0.0490	-0.1730	0.8153	0.0325	1.32E-05	0.6987	5.33E-06	1.0125
205366_s_at	HOXB6	C2	0.2540	-0.0840	0.0485	-0.2459	6.04E-05	0.3496	2.39E-05	0.4317
208887_at	EIF3G	C2	1.13E-04	-0.6371	0.0089	-0.6475	0.6200	0.1338	0.4575	0.2494
211658_at	PRDX2	C2	2.80E-05	-0.9171	0.0064	-0.8453	0.0305	0.7269	0.0078	1.1672
215259_s_at	CADM4	C2	1.40E-04	-1.0293	0.0040	-1.0358	0.0677	-0.7255	0.4234	-0.3458
212261_at	GIGYF2	C2	4.52E-06	-1.2550	0.0086	-1.0074	0.0232	-1.0856	0.0068	-1.5580
213465_s_at	PPP1R7	C2	7.21E-04	-0.5770	0.0038	-0.6889	0.3570	-0.2553	0.6481	-0.1503
220417_s_at	THAP4	C2	6.81E-07	-0.9807	0.0051	-0.7386	0.4780	0.2142	0.5042	0.2353
201007_at	HADHB	C2	1.12E-04	-0.8495	3.47E-05	-1.3178	0.9677	0.0152	0.7090	0.1706
213132_s_at	MCAT	C2	5.58E-04	-0.7196	0.0069	-0.7055	0.4922	0.2341	0.2362	0.4268
201570_at	SAMM50	C2	7.62E-05	-0.7307	4.31E-04	-0.9143	0.3800	0.2601	0.0572	0.6847
204167_at	BTD	C2	1.43E-06	-0.8660	1.56E-04	-0.9428	0.7002	0.1126	0.7544	0.1067
205898_at	CX3CR1	C2	4.11E-06	-0.3360	2.17E-04	-0.3881	0.0603	0.1888	0.3807	0.1111
222314_x_at	EGO	C2	1.64E-05	-0.7399	0.0033	-0.6480	0.2852	-0.2383	0.0716	-0.4901
209239_at	NFKB1	C2	5.40E-04	-0.7243	0.0013	-0.9862	0.5963	0.1581	0.7213	0.1340
215963_x_at	RPL3P7	C2	1.96E-07	-1.1537	0.0039	-0.9575	0.1125	0.5757	0.0834	0.7727
205597_at	SLC44A4	C2	1.84E-04	-0.2651	0.0013	-0.3400	0.0173	0.2861	0.1093	0.2354
210653_s_at	BCKDHB	C2	2.15E-04	-0.5654	0.0063	-0.5749	0.2843	-0.2355	0.6137	-0.1296
214746_s_at	ZNF467	C2	0.5223	0.0853	0.6342	-0.0937	7.90E-07	0.9548	1.91E-06	1.0579
201841_s_at	HSPB1	C2	3.59E-05	0.2972	7.75E-06	0.4776	0.0155	0.2823	0.0098	0.4081
209368_at	EPHX2	C2	6.80E-09	-0.7307	2.91E-05	-0.7370	0.2146	0.2225	0.5187	0.1435
202962_at	KIF13B	C2	6.81E-08	-0.5650	2.04E-05	-0.6882	0.3008	0.1683	0.8830	-0.0315
204015_s_at	DUSP4	C2	6.41E-06	-0.2996	0.0093	-0.2556	0.2732	0.1078	0.4233	0.1046
213082_s_at	SLC35D2	C2	2.25E-05	-0.9101	0.0016	-1.0233	0.0742	-0.6126	0.0983	-0.6503
201004_at	SSR4	C2	3.99E-04	-0.5298	0.0047	-0.6244	0.5394	0.1390	0.0462	0.5381
219939_s_at	CSDE1	C3	3.35E-04	-0.6128	0.0041	-0.6984	0.1210	-0.4683	0.0047	-0.9954
208250_s_at	DMBT1	C3	0.6883	0.1111	0.4819	0.2353	9.80E-05	1.2235	0.0020	1.2764
217742_s_at	WAC	C3	0.0408	-0.3939	0.1477	-0.3826	1.61E-05	-1.3510	2.03E-04	-1.4607
218947_s_at	MTPAP	C3	0.4925	-0.1125	0.4920	-0.1568	6.50E-05	-1.0974	0.0041	-0.9775
203176_s_at	TFAM	C3	0.3035	-0.1318	0.9950	0.0010	7.95E-05	-0.8556	0.0020	-0.7678
208541_x_at	TFAM	C3	0.0343	-0.4456	0.3262	-0.2617	3.93E-05	-1.4818	0.0031	-1.2321
206385_s_at	ANK3	C3	5.28E-04	-0.2268	1.84E-05	-0.3673	0.2113	-0.1459	0.0190	-0.3320
215692_s_at	MPPED2	C3	5.63E-06	0.5087	1.62E-06	0.6278	0.0322	-1.0925	0.0435	-1.1413
203067_at	PDHX	C3	1.10E-04	0.5523	2.62E-04	0.7125	0.3271	0.2344	0.4645	0.2319
210235_s_at	PPFIA1	C3	2.10E-05	0.4289	5.93E-06	0.6325	0.7413	-0.0743	0.4755	-0.2215
202066_at	PPFIA1	C3	6.14E-06	0.4401	7.61E-06	0.6646	0.7597	0.0610	0.6598	-0.1257

Additional file 3. Early or late recurrence associated 216 probe sets (continued)

NAME	Gene symbol	Cluster	Early recurrence-free survival				Late recurrence-free survival			
			Cox-regression		COXPH		Cox-regression		COXPH	
			p-value	coeff.	p-value	coeff.	p-value	coeff.	p-value	coeff.
214073_at	CTTN	C3	9.00E-05	0.3708	9.45E-06	0.5409	0.2026	-0.2377	0.1902	-0.3036
214074_s_at	CTTN	C3	2.86E-05	0.6063	3.20E-07	0.9994	0.8134	-0.0760	0.7182	-0.1456
214782_at	CTTN	C3	6.66E-06	0.5318	9.34E-07	0.7632	0.3719	0.1924	0.7445	0.0892
212754_s_at	MON2	C3	7.18E-04	0.5993	4.59E-04	0.8212	0.6145	0.1524	0.7675	-0.1128
212160_at	XPOT	C3	6.15E-07	0.6435	0.0045	0.5428	0.5870	-0.1255	0.5620	-0.1613
213913_s_at	TBC1D30	C3	3.96E-04	0.4692	6.96E-04	0.6031	0.2772	0.2615	0.4216	0.2450
218604_at	LEMD3	C3	5.56E-08	1.0036	3.21E-06	1.1506	0.9790	0.0087	0.5752	-0.2230
208838_at	CAND1	C3	4.31E-09	0.8674	1.06E-05	0.8336	0.4528	0.2212	0.8685	-0.0570
208839_s_at	CAND1	C3	1.49E-05	0.5922	2.33E-04	0.6305	0.9275	-0.0210	0.2963	-0.2719
202971_s_at	DYRK2	C3	2.54E-06	0.5443	0.0038	0.4490	0.4198	-0.1583	0.2346	-0.2682
220397_at	MDM1	C3	4.95E-06	0.7304	1.17E-06	1.0258	0.0786	0.6749	0.7320	0.2270
218988_at	SLC35E3	C3	4.14E-06	0.5726	2.09E-04	0.5509	0.5041	0.1693	0.4405	-0.3073
201947_s_at	CCT2	C3	6.25E-04	0.4428	0.0014	0.5114	0.3291	0.2135	0.8132	0.0655
201946_s_at	CCT2	C3	9.10E-04	0.3557	0.0017	0.4093	0.8973	0.0259	0.5753	-0.1450
222182_s_at	CNOT2	C3	4.25E-05	0.5764	1.20E-04	0.7176	0.2861	0.2661	0.9846	0.0063
214328_s_at	HSP90AA1	C3	3.14E-05	0.8842	7.25E-05	1.1182	0.4397	-0.2249	0.1735	-0.4219
200884_at	CKB	C3	1.91E-04	0.2702	0.0044	0.2460	0.1414	-0.2437	0.1054	-0.3109
204055_s_at	CTAGE5	C3	3.20E-04	0.7660	0.0085	0.7360	0.4104	0.3021	0.7696	-0.1244
202069_s_at	IDH3A	C3	4.51E-09	0.7967	5.05E-05	0.8067	0.9042	0.0304	0.7379	0.1019
215156_at	WDR61	C3	8.85E-04	0.7842	1.09E-05	1.4080	0.3787	0.3253	0.9963	0.0020
213008_at	FANCI	C3	6.66E-06	0.5020	0.0016	0.4974	0.6826	0.0797	0.4940	0.1659
205464_at	SCNN1B	C3	4.32E-05	0.5319	0.0017	0.5406	0.1355	0.3724	4.74E-04	1.0108
204267_x_at	PKMYT1	C3	5.32E-04	0.8746	0.0016	1.1326	0.1217	0.6248	0.0194	1.1877
204601_at	N4BP1	C3	0.1018	0.3101	0.5548	0.1438	5.12E-05	-1.4013	0.0027	-1.1754
219751_at	SETD6	C3	2.53E-04	0.4998	4.75E-04	0.6790	0.3803	-0.2110	0.9500	0.0179
56821_at	SLC38A7	C3	5.31E-07	1.5029	5.57E-08	2.0704	0.2338	0.6126	0.1096	0.9397
218727_at	SLC38A7	C3	1.43E-06	0.9507	2.08E-06	1.2066	0.0093	0.8203	0.0029	1.0818
209667_at	CES2	C3	0.0091	0.4319	0.0103	0.6109	3.95E-05	1.0980	3.95E-07	1.6261
213509_x_at	CES2	C3	0.0502	0.4224	0.0616	0.5452	1.08E-04	1.2587	1.37E-04	1.4470
204617_s_at	ACD	C3	2.96E-04	0.7189	0.0030	0.7427	0.0477	0.6499	1.29E-04	1.4544
219395_at	ESRP2	C3	6.11E-05	0.5855	9.83E-04	0.7060	0.5438	0.1432	0.0361	0.6534
208785_s_at	MAP1LC3B	C3	6.92E-04	0.5131	0.0049	0.5827	0.6572	0.1079	0.2593	0.3285
220318_at	EPN3	C3	1.43E-05	0.4421	6.16E-04	0.4741	0.5378	0.1242	0.1888	0.2986
205938_at	PPM1E	C3	1.52E-04	0.9555	1.18E-04	1.4005	0.4406	0.4234	0.8959	-0.0957
201890_at	RRM2	C3	1.95E-08	0.4470	0.0010	0.3774	0.1737	0.1723	0.1617	0.2278
210015_s_at	MAP2	C3	0.6018	-0.1112	0.2799	-0.3017	1.04E-04	-1.9271	2.50E-04	-2.2459
218643_s_at	CRIPT	C3	0.1531	0.2659	0.7008	0.1029	1.08E-04	-1.3844	1.05E-04	-1.7200
211573_x_at	TGM2	C3	0.5974	0.1473	0.2118	0.4564	2.17E-05	1.7518	5.20E-05	2.0099
211147_s_at	P2RX6	C3	7.79E-04	-1.4060	0.1131	-0.9681	2.62E-05	2.8117	1.22E-06	4.2866
201519_at	TOMM70A	C3	1.87E-04	0.6804	0.0077	0.6166	0.3115	-0.3111	0.6248	-0.1721

Additional file 3. Early or late recurrence associated 216 probe sets (continued)

NAME	Gene symbol	Cluster	Early recurrence-free survival				Late recurrence-free survival			
			Cox-regression		COXPH		Cox-regression		COXPH	
			p-value	coeff.	p-value	coeff.	p-value	coeff.	p-value	coeff.
213055_at	CD47	C3	0.7018	-0.1167	0.3421	0.3425	1.15E-04	-1.7943	4.06E-04	-1.7991
205930_at	GTF2E1	C3	1.40E-04	0.7190	4.48E-05	1.0827	0.3091	-0.3333	0.0663	-0.7496
213741_s_at	KPNA1	C3	4.84E-04	0.6068	6.74E-04	0.8540	0.8653	0.0523	0.7798	0.1008
201917_s_at	SLC25A36	C3	8.14E-04	0.3382	0.0026	0.5087	0.0044	-0.5328	0.1293	-0.3669
204094_s_at	TSC22D2	C3	2.01E-04	0.4532	0.0072	0.5136	0.6203	-0.0976	0.3857	-0.2106
219507_at	RSRC1	C3	3.02E-05	0.4394	7.81E-04	0.5143	0.0125	-0.4732	0.2229	-0.2702
209380_s_at	ABCC5	C3	4.94E-06	0.4683	0.0028	0.4386	0.0413	0.3449	0.9501	0.0137
202542_s_at	SCYE1	C3	5.01E-04	0.7114	0.0077	0.7174	0.5442	-0.1912	0.4744	-0.2612
215641_at	SEC24D	C3	5.42E-04	0.7606	0.0027	0.8768	0.3338	0.3573	0.9683	0.0193
219158_s_at	NARG1	C3	7.10E-05	0.4480	0.0080	0.3913	3.79E-05	-0.7857	0.0019	-0.6735
208015_at	SMAD1	C3	0.0010	0.5371	0.0013	0.6986	0.0070	0.6835	0.1136	0.5249
204813_at	MAPK10	C3	6.91E-04	0.5829	1.30E-04	0.7704	0.0406	-0.7133	0.0413	-0.8480
208696_at	CCT5	C3	1.09E-04	0.6737	0.0091	0.6451	0.5853	-0.1578	0.8609	0.0626
201736_s_at	MAR_6	C3	7.21E-06	0.6990	4.74E-05	0.9134	0.4250	-0.2163	0.3671	-0.3053
215955_x_at	ARHGAP26	C3	8.14E-04	-1.4475	0.0012	-1.9984	0.5648	0.3756	0.7444	-0.2593
213286_at	ZFR	C3	0.4529	-0.1749	0.5137	0.1933	6.89E-05	-1.6297	6.10E-04	-1.7295
219658_at	PTCD2	C3	6.35E-04	0.6801	4.69E-04	0.8962	0.6028	0.1761	0.9109	0.0419
213793_s_at	HOMER1	C3	4.80E-04	0.3702	1.70E-05	0.6489	0.4064	-0.1378	0.2367	-0.2254
216125_s_at	RANBP9	C3	5.51E-04	0.5732	0.0053	0.5648	0.7675	0.0862	0.5355	0.2068
208496_x_at	HIST1H3G	C3	2.35E-04	0.8540	0.0011	0.9820	0.6189	0.1975	0.7841	-0.1447
209317_at	POLR1C	C3	0.5587	0.1086	0.7250	0.0887	9.99E-05	-1.2442	0.0097	-0.9666
210849_s_at	VPS41	C3	2.07E-06	0.8857	0.0088	0.6454	0.2452	0.3586	0.9836	0.0073
214435_x_at	RALA	C3	3.28E-05	0.5923	0.0060	0.5315	0.0301	-0.4972	0.1902	-0.3501
213577_at	SQLE	C3	1.25E-04	0.3809	0.0032	0.4167	0.1932	-0.2669	0.6142	0.1185
220694_at	DDEF1IT1	C3	5.13E-04	0.5373	0.0010	0.6846	0.3416	0.2464	0.4641	0.2359
219312_s_at	ZBTB10	C3	3.47E-07	0.4281	0.0097	0.2995	0.1058	-0.3535	0.0266	-0.5991
204024_at	OSGIN2	C3	4.70E-06	1.0471	1.23E-04	1.1659	0.2831	-0.4921	0.1343	-0.8414
205694_at	TYRP1	C3	7.85E-04	0.3618	0.0029	0.4571	0.6088	-0.1704	0.7188	-0.1758
211466_at	NFIB	C3	3.48E-04	0.3501	0.0027	0.3853	0.0255	-0.5539	0.0081	-0.7611
202912_at	ADM	C4	1.41E-04	0.2626	0.0047	0.2786	0.9675	0.0052	0.8445	-0.0315
219025_at	CD248	C4	0.9034	0.0205	0.8591	-0.0405	9.89E-05	1.0211	0.0045	0.8945
222101_s_at	DCHS1	C4	0.6020	0.0661	0.9062	-0.0222	4.29E-05	0.8536	4.32E-04	0.9600
204575_s_at	MMP19	C4	0.6901	0.0801	0.3013	0.2599	1.05E-04	1.0439	0.0055	0.9831
204682_at	LTBP2	C4	0.3835	0.0956	0.2862	0.1625	2.95E-05	0.7606	5.10E-04	0.7431
203940_s_at	VASH1	C4	0.8983	0.0226	0.4594	-0.1764	7.79E-05	1.1454	0.0031	1.0111
203151_at	MAP1A	C4	0.1290	0.3265	0.1569	0.4088	5.76E-05	1.1798	7.26E-04	1.2441
214684_at	MEF2A	C4	5.83E-04	0.5383	0.0065	0.5486	0.3360	-0.2587	0.1048	-0.5208
213725_x_at	XYLT1	C4	4.15E-04	0.4127	0.0014	0.5646	0.1656	0.2881	0.2649	0.3113
214761_at	ZNF423	C4	0.1764	-0.1415	0.1970	-0.1905	3.21E-05	0.6696	2.37E-04	0.8055
219478_at	WFDC1	C4	1.33E-06	0.6270	0.0031	0.4825	0.4682	0.2085	0.3749	0.2875

Additional file 3. Early or late recurrence associated 216 probe sets (continued)

NAME	Gene symbol	Cluster	Early recurrence-free survival				Late recurrence-free survival			
			Cox-regression		COXPH		Cox-regression		COXPH	
			p-value	coeff.	p-value	coeff.	p-value	coeff.	p-value	coeff.
202310_s_at	COL1A1	C4	0.3452	0.0712	0.3764	0.0963	9.63E-06	0.6326	0.0044	0.5270
215076_s_at	COL3A1	C4	0.6664	0.0387	0.9771	0.0037	1.54E-05	0.7572	0.0070	0.6028
201438_at	COL6A3	C4	0.5774	0.0549	0.9478	0.0086	5.01E-05	0.7539	0.0049	0.6048
209099_x_at	JAG1	C4	0.0533	0.2229	0.2071	0.2126	1.06E-05	0.8747	3.48E-04	0.9283
216268_s_at	JAG1	C4	0.0274	0.2335	0.1930	0.2035	6.17E-05	0.7206	8.64E-04	0.8085
218559_s_at	MAFB	C4	0.4451	0.0909	0.5267	0.1004	6.03E-05	0.9048	0.0029	0.8034
202619_s_at	PLOD2	C4	3.05E-04	0.2861	0.0033	0.3091	0.2850	-0.1295	0.0943	-0.2347
202620_s_at	PLOD2	C4	9.30E-07	0.3719	0.0037	0.2980	0.5433	-0.0666	0.3198	-0.1291
210135_s_at	SHOX2	C4	8.28E-05	0.4592	0.0061	0.5109	0.0269	0.4916	0.1524	0.3920
213519_s_at	LAMA2	C4	0.0112	-0.2625	0.0572	-0.2787	9.62E-05	0.6384	0.0031	0.5854
210517_s_at	AKAP12	C4	0.9138	-0.0109	0.8655	0.0232	4.35E-05	0.6346	0.0022	0.5545
219434_at	TREM1	C4	5.85E-04	0.5863	0.0045	0.5668	0.6462	0.1641	0.5112	0.2825
213943_at	TWIST1	C4	2.52E-04	0.2891	1.92E-04	0.3662	0.3907	0.1405	0.2205	0.2475
209960_at	HGF	C4	0.6017	-0.0961	0.5910	-0.1530	1.43E-06	1.0188	0.0028	0.9274
202404_s_at	COL1A2	C4	0.2895	0.0892	0.5585	0.0648	6.01E-05	0.6525	0.0087	0.5088
203477_at	COL15A1	C4	0.5737	0.0510	0.5747	-0.0722	7.94E-05	0.6479	0.0037	0.5857
