# Supplementary material for: A signature of epithelial-mesenchymal plasticity and stromal activation in primary tumor modulates late recurrence in breast cancer independent of disease subtype
Source: Breast Cancer Res. 2014 Jul 25;16:407. doi: 10.1186/s13058-014-0407-9 (PMC4187325; doi:10.1186/s13058-014-0407-9)
Supplement: Supplementary file 4 — Additional file 4: Significant pathways in selected gene sets. Table of top activated pathways of selected gene sets. (PDF 6 KB) [file 13058_2014_407_MOESM4_ESM.pdf]

Additional file 4. Significant pathways in selected gene sets

Gene set	Maps	FDR	Network Objects	Process
**Cluster C2**	Development_PEDF signaling	0.0327	NFKB1	Anti-apoptosis; Immune reponse
	LRRK2 in neurons in Parkinson's disease	0.0954	AP2A2,	
	Immune response_Inflammasome in inflammatory response	0.0954	NFKB1, PYCARD	Inflammatory response
	Signal transduction_Erk Interactions: Inhibition of Erk	0.0954	DUSP4,	Regulation of ERK-dependent pathways
	Apoptosis and survival_APRIL and BAFF signaling	0.0954	NFKB1	Inflammation
**Cluster C3**	ATP/ITP metabolism	0.1656	POLR1C, RRM2	
	Oxidative stress_Role of ASK1 under oxidative stress	0.2301	HSP90AA1, MAPK10, RRM2	Microtubule destabilization
	Development_Regulation of telomere length and cellular immortalization	0.2301	ACD, HSP90AA1	Cellular immortalization
	Immune response_HMGB1 release from the cell	0.2301	MAPK10, KPNA1,	Inflammatory response
	Apoptosis and survival_TNF-alpha-induced Caspase-8 signaling	0.2301	HSP90AA1	Degradation in the 26S proteasome
**Cluster C4**	Development_Regulation of epithelial-to-mesenchymal transition (EMT)	0.0012	JAG1, HGF, TWIST1	Activation of EMT
	Development_HGF-dependent inhibition of TGF-beta-induced EMT	0.0036	HGF	Inhibition of EMT
	Cell adhesion_PLAU signaling	0.0037	HGF	
	Development_PIP3 signaling in cardiac myocytes	0.0037	HGF	
	Development_HGF signaling pathway	0.0037	HGF	Inhibition of EMT
**51-gene**	Cell adhesion_ECM remodeling	0.0020	COL1A1, COL1A2, COL3A1, ITGA5, LAMA4, LAMB1, VCAN	ECM remodeling
	Immune response_IL-13 signaling via PI3K-ERK	0.0020	COL1A2, CALM1, IL13RA1, TNC	Fibrosis; Inflammation; Inhibition of oxyen and reactive oxygen species production
	Development_TGF-beta-dependent induction of EMT via SMADs	0.0114	JAG1, SNAI2, TWIST1	EMT
	Development_TGF-beta-dependent induction of EMT via RhoA, PI3K and ILK.	0.0136	LIMS1, SNAI2, TGFB1I1	EMT
	Development_Endothelin-1/EDNRA transactivation of EGFR	0.0136	ADAM9, COL1A1, COL1A2, CALM1	Remodeling / Fibrosis
