# Supplementary material for: A signature of epithelial-mesenchymal plasticity and stromal activation in primary tumor modulates late recurrence in breast cancer independent of disease subtype
Source: Breast Cancer Res. 2014 Jul 25;16:407. doi: 10.1186/s13058-014-0407-9 (PMC4187325; doi:10.1186/s13058-014-0407-9)
Supplement: Supplementary file 5 — Additional file 5: Forty-eight probe sets that were up/downregulated in the stroma of subgroup G4. Table of 48 probe sets that were up/downregulated in the stroma of subgroup G4. (PDF 35 KB) [file 13058_2014_407_MOESM5_ESM.pdf]

Additional file 5. 48 probe sets that were up/down regulated in stroma of Subgroup G4

NAME	Gene symbol	T-test (FDR)				Correlation
		G4 vs. G1	G4 vs. G2	G4 vs. G3	G4 vs. G1&G2&G3	
217945_at	BTBD1	0.431	0.430	0.033	0.011	Upregulated in G4
214085_x_at	GLIPR1	0.142	0.423	0.016	0.013	Upregulated in G4
210609_s_at	TP53I3	0.198	0.423	0.008	0.017	Upregulated in G4
218974_at	SOBP	0.142	0.600	0.045	0.020	Upregulated in G4
201739_at	SGK1	0.142	0.461	0.038	0.020	Upregulated in G4
218803_at	CHFR	0.162	0.505	0.025	0.020	Upregulated in G4
213728_at	LAMP1	0.142	0.423	0.038	0.020	Upregulated in G4
217975_at	WBP5	0.222	0.634	0.016	0.020	Upregulated in G4
204209_at	PCYT1A	0.341	0.585	0.008	0.023	Upregulated in G4
205399_at	DCLK1	0.142	0.486	0.054	0.026	Upregulated in G4
207002_s_at	PLAGL1	0.142	0.505	0.036	0.026	Upregulated in G4
208961_s_at	KLF6	0.248	0.442	0.066	0.026	Upregulated in G4
36030_at	IFFO1	0.249	0.434	0.038	0.026	Upregulated in G4
215640_at	TBC1D2B	0.236	0.423	0.074	0.026	Upregulated in G4
201300_s_at	PRNP	0.224	0.616	0.025	0.026	Upregulated in G4
219974_x_at	ECHDC1	0.204	0.486	0.066	0.036	Upregulated in G4
212585_at	OSBPL8	0.447	0.506	0.033	0.038	Upregulated in G4
204955_at	SRPX	0.392	0.509	0.054	0.040	Upregulated in G4
219025_at	CD248	0.200	0.430	0.066	0.040	Upregulated in G4
212687_at	LIMS1	0.142	0.477	0.037	0.040	Upregulated in G4
203909_at	SLC9A6	0.186	0.430	0.054	0.040	Upregulated in G4
205745_x_at	ADAM17	0.409	0.597	0.020	0.043	Upregulated in G4
209045_at	XPNPEP1	0.142	0.509	0.135	0.046	Upregulated in G4
202501_at	MAPRE2	0.146	0.506	0.100	0.046	Upregulated in G4
218677_at	S100A14	0.318	0.423	0.031	0.015	Downregulated in G4
210243_s_at	B4GALT3	0.710	0.477	0.016	0.020	Downregulated in G4
206546_at	SYCP2	0.386	0.524	0.083	0.020	Downregulated in G4
208644_at	PARP1	0.263	0.434	0.066	0.021	Downregulated in G4
202731_at	PDCD4	0.241	0.465	0.107	0.021	Downregulated in G4
202209_at	LSM3	0.391	0.608	0.031	0.021	Downregulated in G4
220277_at	CXXC4	0.344	0.640	0.054	0.023	Downregulated in G4
215691_x_at	HSPB11	0.343	0.430	0.054	0.026	Downregulated in G4
218270_at	MRPL24	0.315	0.423	0.054	0.026	Downregulated in G4
207877_s_at	NVL	0.355	0.430	0.108	0.026	Downregulated in G4
201966_at	NDUFS2	0.683	0.423	0.036	0.028	Downregulated in G4
220302_at	MAK	0.215	0.465	0.026	0.029	Downregulated in G4
202730_s_at	PDCD4	0.249	0.537	0.162	0.035	Downregulated in G4
202597_at	IRF6	0.226	0.423	0.085	0.037	Downregulated in G4
219121_s_at	ESRP1	0.278	0.430	0.112	0.038	Downregulated in G4
201897_s_at	CKS1B	0.731	0.423	0.074	0.040	Downregulated in G4

Additional file 5. 48 probe sets that were up/down regulated in stroma of Subgroup G4 (continued).

NAME	Gene symbol	T-test (FDR)				Correlation
		G4 vs. G1	G4 vs. G2	G4 vs. G3	G4 vs. G1&G2&G3	
201999_s_at	DYNLT1	0.676	0.640	0.025	0.040	Downregulated in G4
219041_s_at	REPIN1	0.163	0.506	0.066	0.040	Downregulated in G4
219298_at	ECHDC3	0.362	0.672	0.059	0.040	Downregulated in G4
208822_s_at	DAP3	0.433	0.430	0.083	0.046	Downregulated in G4
203073_at	COG2	0.286	0.505	0.271	0.046	Downregulated in G4
202488_s_at	FXYD3	0.392	0.505	0.066	0.046	Downregulated in G4
214170_x_at	FH	0.409	0.572	0.066	0.047	Downregulated in G4
205464_at	SCNN1B	0.508	0.505	0.104	0.049	Downregulated in G4
