# Supplementary material for: A signature of epithelial-mesenchymal plasticity and stromal activation in primary tumor modulates late recurrence in breast cancer independent of disease subtype
Source: Breast Cancer Res. 2014 Jul 25;16:407. doi: 10.1186/s13058-014-0407-9 (PMC4187325; doi:10.1186/s13058-014-0407-9)
Supplement: Supplementary file 6 — Additional file 6: Probe sets of 51-gene signature of stromal activation in primary tumor. Table of probe sets from 51-gene signature of stromal activation in primary tumor. (PDF 36 KB) [file 13058_2014_407_MOESM6_ESM.pdf]

Additional file 6. Probe sets of 51-gene signature of stromal activation in primary tumor.

NAME	Gene	Correlation with SPC1 Pearson coefficient (R)	G4 vs. G1&G2&G3 T-test (FDR), n=46	Cox-regression survival (n=743)	
				p-value	coeff.
204362_at	SKAP2	0.6942	0.0051	0.0063	0.2683
212687_at	LIMS1	0.6656	0.0075	7.81E-04	0.4758
214081_at	PLXDC1	0.5929	0.0205	0.0033	0.3459
218559_s_at	MAFB	0.5843	0.0099	0.0062	0.2876
210135_s_at	SHOX2	0.5843	0.0334	6.00E-06	0.4661
220244_at	LOH3CR2A	0.5750	0.0341	8.44E-04	0.4581
202237_at	NNMT	0.5724	0.0037	0.0089	0.1922
201645_at	TNC	0.5707	0.0494	0.0037	0.1493
37408_at	MRC2	0.5690	0.0013	0.0079	0.3103
202202_s_at	LAMA4	0.5667	3.75E-04	2.25E-04	0.3194
202968_s_at	DYRK2	0.5631	0.0252	6.78E-05	0.6001
213169_at	SEMA5A	0.5596	0.0331	0.0083	0.2808
208443_x_at	SHOX2	0.5464	0.0280	6.50E-05	0.6088
213139_at	SNAI2	0.5446	0.0214	0.0092	0.1798
212887_at	SEC23A	0.5381	4.13E-04	5.48E-04	0.3228
212334_at	GNS	0.5374	0.0453	2.70E-04	0.5010
201185_at	HTRA1	0.5358	0.0198	0.0061	0.2144
220694_at	DDEF1IT1	0.5286	0.0354	5.57E-04	0.4588
201505_at	LAMB1	0.5284	2.84E-05	0.0056	0.1896
212488_at	COL5A1	0.5281	0.0194	0.0031	0.1864
209099_x_at	JAG1	0.5278	0.0171	8.17E-05	0.3920
202311_s_at	COL1A1	0.5246	0.0263	0.0041	0.1761
204163_at	EMILIN1	0.5184	0.0015	1.33E-04	0.4195
209780_at	PHTF2	0.5161	0.0049	4.44E-04	0.4639
212489_at	COL5A1	0.5062	0.0287	0.0034	0.1862
211709_s_at	CLEC11A	0.5061	0.0210	0.0078	0.2568
216268_s_at	JAG1	0.5004	0.0176	7.21E-05	0.3611
213001_at	ANGPTL2	0.4972	0.0323	0.0024	0.2711
215076_s_at	COL3A1	0.4934	5.63E-04	0.0091	0.2115
45749_at	FAM65A	0.4902	0.0047	0.0032	0.8000
201438_at	COL6A3	0.4858	0.0106	0.0078	0.2343
213869_x_at	THY1	0.4850	0.0030	0.0015	0.3329
203688_at	PKD2	0.4845	0.0057	0.0026	0.3290
204114_at	NID2	0.4749	0.0378	7.22E-04	0.2754
221731_x_at	VCAN	0.4684	0.0262	0.0050	0.2039
209651_at	TGFB1I1	0.4600	0.0292	1.26E-04	0.3381
200653_s_at	CALM1	0.4594	1.17E-04	0.0088	0.2697
212977_at	CXCR7	0.4528	0.0205	3.30E-05	0.2418
210904_s_at	IL13RA1	0.4442	0.0443	0.0014	0.3319
202404_s_at	COL1A2	0.4421	0.0120	0.0021	0.2313
208851_s_at	THY1	0.4411	0.0243	0.0029	0.3171

Additional file 6. Probe sets of 51-gene signature of stromal activation in primary tumor (continued)

NAME	Gene	Correlation with SPC1 Pearson coefficient (R)	G4 vs. G1&G2&G3 T-test (FDR), n=46	Cox-regression survival (n=743)	
				p-value	coeff.
212522_at	PDE8A	0.4265	0.0022	0.0048	0.3419
212930_at	ATP2B1	0.4140	0.0122	0.0028	0.3422
201389_at	ITGA5	0.4138	0.0474	3.37E-05	0.6409
203573_s_at	RABGGTA	0.4053	0.0430	0.0015	0.6240
203835_at	LRRC32	0.3992	0.0251	0.0071	0.3641
202284_s_at	CDKN1A	0.3976	0.0369	0.0039	0.3233
210809_s_at	POSTN	0.3842	0.0309	0.0032	0.2066
213943_at	TWIST1	0.3838	0.0042	3.17E-04	0.2593
215716_s_at	ATP2B1	0.3741	0.0176	0.0074	0.2685
202122_s_at	M6PRBP1	0.3697	0.0228	0.0025	0.4762
202381_at	ADAM9	0.3430	0.0135	0.0020	0.2135
202971_s_at	DYRK2	0.3390	0.0409	4.94E-04	0.3454
201655_s_at	HSPG2	0.3231	0.0042	0.0017	0.3413
214196_s_at	TPP1	0.3026	0.0069	9.13E-04	0.4392
201792_at	AEBP1	0.2893	0.0341	0.0056	0.1981
221997_s_at	MRPL52	0.2701	0.0477	0.0057	0.6397
